# Applicability and precautions of use of liver injury biomarker FibroTest. A reappraisal at 7 years of age

**DOI:** 10.1186/1471-230X-11-39

**Published:** 2011-04-14

**Authors:** Thierry Poynard, Mona Munteanu, Olivier Deckmyn, Yen Ngo, Fabienne Drane, Djamila Messous, Jean Marie Castille, Chantal Housset, Vlad Ratziu, Françoise Imbert-Bismut

**Affiliations:** 1 APHP UPMC Paris Liver Center, Paris, France; 2 Biopredictive, Paris, France

## Abstract

**Background:**

FibroTest (FT) is a validated biomarker of fibrosis. To assess the applicability rate and to reduce the risk of false positives/negatives (RFPN), security algorithms were developed. The aims were to estimate the prevalence of RFPN and of proven failures, and to identify factors associated with their occurrences.

**Methods:**

Four populations were studied: 954 blood donors (P1), 7,494 healthy volunteers (P2), 345,695 consecutive worldwide sera (P3), including 24,872 sera analyzed in a tertiary care centre (GHPS) (P4). Analytical procedures of laboratories with RFPN > 5% and charts of P4 patients in with RFPN were reviewed.

**Results:**

The prevalence of RFPN was 0.52% (5/954; 95%CI 0.17-1.22) in P1, 0.51% (38/7494; 0.36-0.70) in P2, and 0.97% (3349/345695; 0.94-1.00) in P3. Three a priori high-risk populations were confirmed: 1.97% in P4, 1.77% in HIV centre and 2.61% in Sub-Saharan origin subjects. RFPN was mostly associated with low haptoglobin (0.46%), and high apolipoproteinA1 (0.21%). A traceability study of a P3 laboratory with RFPFN > 5% permitted to correct analytical procedures.

**Conclusion:**

The mean applicability rate of Fibrotest was 99.03%. Independent factors associated with the high risk of false positives/negatives were HIV center, subSaharan origin, and a tertiary care reference centre, although the applicability rate remained above 97%.

## Background

Due to the limitations of liver biopsy biomarkers are widely used as a non-invasive alternative in patients with chronic liver disease to assess fibrosis stage and necroinflammatory activity [1-3]. One of the most validated serum biomarkers, Fibrotest-Actitest (FT-AT), was introduced on the market in September 2002 and has been widely prescribed since then [2-4]. The French Heath Authorities (HAS) have recommended the following precautions of use for FT: (1) the laboratory that performs the test must use the appropriate assay technique and ensure proper quality control (e.g. with regard to sample storage), and (2) the person who prescribes the test must consider confounding factors when interpreting test results. Patients should have no intercurrent illness, in particular acute inflammation, hemolysis, or Gilbert's syndrome, and should be taking no medications that are known to cause elevated bilirubin levels [3].

The aim of the "precautions of use" is to reduce the number of false positive/false negative. The purest definition of false positive/negative for a biomarker of liver injury can be obtained only by large surgical biopsy [5,6]. Therefore there is no perfect reference test for the definition of false positive/negative in a large population. From several studies of discordances results between biopsy (the classical reference) and FT, the prevalence of discordant results is around 25%, half of the cases being due to biopsy failure and half being due to FT failure [4,5,7-9].

Due to these limitations of the classical definition of false positive/false negative, we propose to use the concept of "high risk profile of false positive/false negative results" (RFPN) and to use it for the definition of FT applicability for the identification of deviance from recommended pre-analytical and analytical procedures [10-15].

"Security algorithms" were elaborated in order to identify subjects with RFPN. These algorithms were initially derived from the first validation of FT-AT [8,16-20]. FT-AT are calculated through a centralized website http://www.biopredictive.com after entering the results of the panel's components [4]. Therefore it has been possible to identify RFPN during this step and the suspected RFPN components are indicated on the results sheets.

In order to improve the medical service, the specific aims of the present study were to estimate the applicability of FT using prevalence of RFPN and of proven failures, to identify factors associated with their occurrence and to identify new causes. The integrated database of 354,143 tests, which is the accumulation of the first seven years of FT-AT prescription, was used.

## Methods

### Populations included

**Figure 1:**
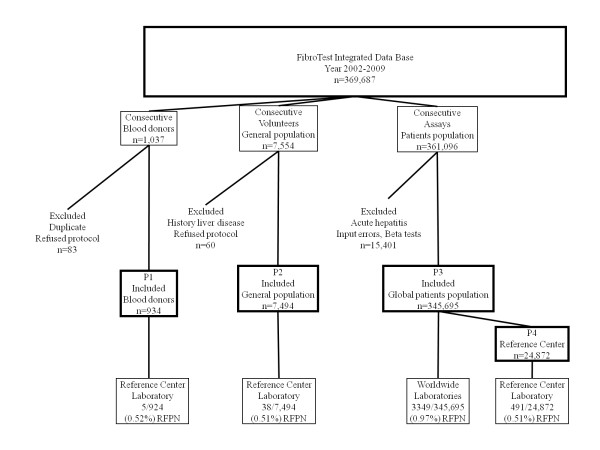
**Flow sheet of included populations**.

Four prospective populations were included (Figure 1): 954 blood donors between January 2003 and March 2004 (P1); 7,494 healthy volunteers from a general population without previous history of liver disease between June 2006 and September 2008 (P2); 345,695 consecutive FT-AT (P3) analyzed on the dedicated website between October 2002 and June 2009, including 24,872 patients with clinical characteristics seen in a tertiary care reference centre, Groupe Hospitalier Pitié Salpêtrière, Paris, France (reference population P4), for which the detailed clinical characteristics were easier to retrieve. The components of FT-AT were analyzed on fresh samples; the same laboratory (reference center) was used for P1, P2 and P4; the P3 components of FT-AT were analyzed prospectively in 449 labs in 35 countries.

### Device description

The FibroTest is comprised of two parts: 1) biomarker assays (components of the panel) measuring alpha2-macroglobulin (A2M), apolipoprotein A1 (ApoA1), haptoglobin, gamma-glutamyl-transpeptidase (GGT) and total bilirubin (bilirubin); and 2) a software containing a fixed, pre-determined algorithm to generate the FibroTest score from the components, adjusted for age and gender.

To be validated and interpretable, the components assays of FT must follow the pre-analytical and analytical recommendations: measurements are calibrated and performed according to standardized reagents against reference materials; expression in multiples of the upper limit of reference values should not be employed [10-15]; and company-approved analyzers and kits are used to generate quantified values of the individual markers [4]. Since the first study, 157 peer-reviewed publications including several meta-analyses, have consistently validated the accuracy of FT-AT for assessing the stages of liver fibrosis when these technical recommendations have been utilized and when the area under the receiver operating characteristics curve has been standardized according to stage spectrum. [Additional file 1].

### Endpoints

The main endpoint was RFPN, the percentage of patients with values outside the reference ranges (abnormal values) and in whom the switch to the median value of the given abnormal variation component induced a variation of at least 0.30 of the FT value. This variation was considered clinically significant, as a variation of 0.30 in FT is equivalent to 1.5 histological METAVIR score of fibrosis [21]. Abnormal values of each component were defined as those beyond the 98% percentile of the normal distribution (one lower percentile or one upper percentile). The reference ranges for each component were established from the normal distribution observed in the reference laboratory (Biochemistry Department, Groupe Hospitalier Pitié Salpêtrière, Paris France) during the first studies in patients with HCV [16], HBV [17], alcoholic liver disease [18], and non-alcoholic fatty liver disease [19] [Additional file 2 Table S1, Additional file 2 Table S2, Additional file 2 Table S3, Additional file 2 Table S4]. These "security" algorithms were previously validated using analyses of discordances versus liver biopsy [7] and discordances with liver stiffness measurements [8,9].

The specificity of FT-AT was checked using the P1 and P2 control populations after the exclusion of cases with a previous history of liver diseases and exclusion of RFPN. In these controls, the prevalence of presumed advanced fibrosis (FT > 0.48, equivalent to METAVIR stage F2F3F4) or advanced activity (AT > 0.52, equivalent to METAVIR grade A2A3) [21] was assumed to be lower than 5% in both groups. P2 subjects with FT suggesting advanced fibrosis were prospectively retested in the reference center [22].

### High-risk related factors analyzed

The risk factors associated with RFPN were assessed in each population. Two categories of factors were considered: analytical, and non-analytical.

Charts of the RFPN in the P4 group were re-analyzed retrospectively by three experts (TP, MM and YN) in order to identify new possible causes of component errors and to validate the positive predictive value of high-risk profiles. In each case the cause of failure was attributed to FT or not according to the a priori (pre-determined) following rules: the disease was advanced fibrosis (METAVIR stage F2F3F4); biopsy was the reference if performed less than 5 years apart; when no biopsy had been performed but an LSM was interpretable (at least 10 measures, success rate greter than 60% and interquartile range lower than 30%), it was taken as reference (advanced fibrosis if greater than 7.1 kPa); when esophageal varices or ascites were present, it was interpreted as advanced fibrosis; for low haptoglobin, if there was no reference but a cause of hemolysis was identified, the FT > = 0.48 was considered a false positive. When no reference presented with a clear cause of component error (such as hemolysis for haptoglobin or severe undernutrition for A2M or ApoA1), the case was stated to be indeterminate.

#### Analytical factors

The impact of analytical factors was assessed using three methods, as performed on the P3.

First, we tested the a priori hypothesis that the analytical procedures improved with time, with a decrease in RFPN in the last 3 years of this cohort (median).

Second, we tested the a priori hypothesis that the prevalence of RFPN could be lower in laboratories that performed more FTs than those that performed less, the cut-off being chosen as 10,000 for the 7 years of follow-up, as these 6 laboratories represented 50% of the overall assays.

Thirdly, the analytical procedures of laboratories with a prevalence of RFPN > 5% were reviewed prospectively during the follow-up to check whether the pre-analytical and analytical recommendations had been followed.

#### Non-analytical factors

Known risk factors were those mentioned by Health Authorities: acute inflammation, hemolysis, Gilbert's syndrome and medications associated with elevated bilirubin [3]. Suspected risk factors were those mentioned in publications: ethnicity, HIV (including cholestatic anti viral drugs), large ascite and undernutrition [3,4,8,22-24].

We therefore predetermined 3 types of populations concerning RFPN: a low-risk group (P1 blood donors and P2 healthy volunteers), an intermediate-risk group (P3 patients investigated for chronic liver diseases, and three high risk groups: tertiary care reference centre (P4), HIV centre and patients from sub-Saharan origin.

In the P2, P3 and P4 groups, we tested the hypothesis that component variability could be associated with ethnicity. We therefore analyzed the association between RFPN and the following areas of residency: Western Europe, the Middle East, Eastern Europe, North Africa, North America, Central America, and the Far East. In the P2 group we tested the pre-determined hypothesis that subjects living in SubSaharan Africa should have an increased risk of RFPN associated with abnormally low haptoglobin levels (haptoglobin polymorphism with anahaptoglobinemia) [24].

In the P3 group, the impact of coinfection with HIV was assessed by comparing one center that assess FT-AT almost exclusively in coinfected patients with HIV and HCV or HBV with other centers that were not specialized in HIV [23].

The reference centre is a tertiary care centre where very high-risk RFPN patients were screened for FT, such as patients with severe undernutrition and sepsis. In the P4 group, charts were reviewed for the usual causes of RFPN: extra-hepatic cholestasis patients (abnormal increase in GGT and bilirubin); hemolysis (abnormal decrease in haptoglobin); Gilbert's syndrome (abnormal increase in bilirubin); acute inflammation, i.e. acute sepsis (abnormal increase in haptoglobin); and severe undernutrition with total proteins < 50 g/L.

Patients at high-risk related to acute hepatitis were supposed to be detected by extreme values of ALT, greater than 622 IU/L (1% upper percentile observed in first publications), and were excluded in this study, which focused on chronic liver diseases.

### Statistical methods

Comparisons used the Student's t-test for univariate analysis and logistic regression analysis for multivariate analysis. NCSS statistical software was used [25].

To reduce the risk of type 1 error due to multiple testing (five pre-determined risk factors, two covariates, four populations and five FT components) only p-values less than or equal to 0.0001 were considered to be significant. Multivariate analysis included age and gender as covariates.

The present study was in compliance with the Helsinki Declaration, was an epidemiological study, not an interventional study and did not require the approval of an ethical committee. For the population 4, patients hospitalized in the "Groupe Hospitalier Pitié Salpêtrière", and for which we looked to the possible causes of false positives or false negatives, the protocol was recognized as not interventional by the Ethical Committee (November 25th 2005). All the data were strictly anonymous data, the database was declared to the French authorities "Commission Informatique et Liberté", in accordance with the French law on information processing,

## Results

### Populations included

The characteristics of the included populations are given in Table 1. As expected, the P1 population, made up of blood donors, was younger than the others. The P2 group, a population representative of the French population older than 40 years [22], was older than the P3 group, the worldwide population. The majority of FT-AT assays were performed in Western Europe (86%), followed by the Middle East (8.10%). The P4 population, the reference centre, had more males, more residents of Western Europe and longer laboratory experience in FT-AT testing compared with the P3 population.

**Table 1:** Characteristics of included populations

Name of Population	P1 Blood donors	P2 Healthy volunteers	P3 Worldwide patients	P4 Reference Center patients
**Consecutive cases (n)**	**1,037**	**7,554**	**361,096**	**24,872**

**Included cases (n)**	**n = 954**	**n = 7,494**	**n = 345,695**	**n = 24,872**

**Age mean year (SD; range)**	35.75 (12.24;18-67)	56.97 (6.77; 40-90)	49.73 (13.87; 0-101)	49.36 (13.60;0-101)
**Gender**				
Male	483 (50.63%)	4138 (55.22%)	198612 (57%)	15530 (62.44%)
Female	471 (49.37%)	3356 (44.78%)	147083 (43%)	9342 (37.56%)
**Country of residency/ancestry**	**Residency**	**Ancestry**	**Residency**	**Residency**
Western Europe	1037 (100%)	6705 (89.47%)	295685 (85.53%	24,872 (100%)
Middle East	0 (0%)	0 (0%)	28001 (8.10%)	0 (0%)
Eastern Europe	0 (0%)	0 (0%)	5209 (1.51%)	0 (0%)
North Africa	0 (0%)	467 (6.23%)	6464 (1.87%	0 (0%)
North America	0 (0%)	0% (0%)	7726 (2.23%	0 (0%)
Central America	0 (0%)	0% (0%)	1436 (0.42%)	0 (0%)
Far East	0 (0%)	92 (1.23%)	993 (0.29%)	0 (0%)
South America	0 (0%)	0% (0%)	181 (0.05%)	0 (0%)
Sub-Saharan Africa	0 (0%)	230 (3.07%)	0 (0%)	0 (0%)
**Laboratory experience**				
> 10,000 tests (n = 6)	954 (100%)	7494 (100%)	143772 (41.49%)	24,872 (100%)
< 10,000 tests (n = 443)	0 (0%)	0 (0%)	201923 (58.41%)	0 (0%)
**HIV positive patients**				
Yes	0 (0%)	2 (0.003%)	2602 (0.75%)^3^	NA
Negative	954 (100%)	1012/1014 (13.50%)	0 (0%)	NA
Unknown	0 (0%)	6443 (66.50%)	343093 (99.25%)	NA
**Liver Disease Risk**			Unknown	Only known for high risk profile
Non Alcoholic Fatty Liver	105 (11.01%)	2930 (39.29%)	NA	NA
Alcoholic Liver Disease	82 (8.59%)	883 (11.84%)	NA	NA
NAFLD/ALD	19 (1.99%)	1036 (13.89%)	NA	NA
Hepatitis C Virus	0 (0%)	31 (0.42%)	NA	NA
Hepatitis B Virus	0 (0%)	3 (0.04%)	NA	NA
Other	0 (0%)	2 (0.03%)	NA	NA
No Risk	748 (78.41%)	2572 (34.49%)	NA	NA
**FibroTest**				
FibroTest mean (SD; 1%-99% percentiles)	0.11 (0.08;0.01-0.41)	0.17 (0.12;0.03-0.60)	0.42 (0.27;0.03-0.97)	0.40 (0.28;0.03-0.98)
F0 or F0-F1	908 (95.18%)	6302 (84.09)%	131658 (38.09%)	10526 (42.32%)
F1 or F1-F2	42 (4.40%)	927 (12.37)%	77974 (22.56%)	5302 (21.32%)
F2	0 (0%)	151 (2.01%)^2^	31646 (9.15%)	2059 (8.28%)
F3 or F3-F4	3 (0.31%)^1^	76 (1.01%)^2^	46021(13.31%)	2979 (11.98%)
F4	1 (0.10%)^1^	38 (0.51%)^2^	58396 (16.89%)	4006 (16.11%)
**Actitest**				
Actitest mean (SD; 1%-99% percentiles)	0.08 (0.07;0.01-0.40)	0.11 (0.10;0.02-0.52)	0.37 (0.26;0.02-0.95)	0.30 (0.24;0.02-0.93)
A0 or A0-A1	935 (98.01%)	7060 (94.52%)	166068 (48.05%)	14823 (59.72%)
A1 or A1-A2	16 (1.68%)	347 (4.46%)	84102 (24.33%)	5207 (20.98%)
A2 or A2-A3	1 (0.10%)	39 (0.48%)	26714 (7.73%)	1455 (5.86%)
A3	2 (0.21%)	48 (0.64%)	68741 (19.89%)	3334 (13.43%)
**FibroTest-ActiTest**				
Interpretable	949 (99.48%; 98.78-99.83)	7456 (99.49%; 99.30-99.64)	342,346 (99.03%; 99.00-99.06)	24381 (98.03%; 97.95-98.20)
High risk False Positive/Negative (95% CI)	5 (0.52%; 0.17-1.22)	38 (0.51%; 0.36-0.70)	3349 (0.97%; 0.94-1.00)	491 (1.97%; 1.80-2.15)

^1 ^All four cases with Fibrotest greater than 0.48 had a high-risk profile of components which were detected by security algorithms (one low A2M and 3 low haptoglobin).

^2 ^31 cases out of the 265 cases with FibroTest greater than 0.48 had a high-risk profile of components which were detected by security algorithms.

NA = not available

^3 ^Hospital unit with prevalence of HIV > 90%

### Normal values

In comparison with initial studies, FT references values were confirmed in the two populations without a history of liver disease: the mean FT in P1 = 0.11 (98% percentiles 0.01-0.41); in P2, FT = 0.17 (0.02-0.52). Only 2.75% (3/109; 95% CI 0.57-7.8) of P2 subjects that were reinvestigated for FT > 0.48 were proven to be false positive, i.e., 0.04% (3/7, 554; 0.01-0.12) proven false positive rate.

### Prevalence of RFPN

According to the populations, the prevalence of RFPN was 0.52% (5/954; 95% CI 0.17-1.22) in P1 and 0.51% (38/7494; 95% CI 0.36-0.70) in P2 (blood donors and general population, two usually low-risk populations); 0.97% (3349/345695; 95% CI 0.94-1.00) in P3 (intermediate risk); and 1.97% (491/24872; 95% CI 1.80-2.15) in P4, a high-risk population (Table 1). This RFPN prevalence in P4 was significantly higher (P < 0.0001) than in the P3 worldwide population, confirming the a priori high-risk of a tertiary care reference centre. There was also a higher RFPN prevalence versus P3 (P < 0.0001) in the two other high-risk populations: in patients of the HIV centre, the RFPN prevalence was 1.77% (46/2606; 95% CI 1.30-2.35), and in subjects of sub-Saharan origin it was 2.61% (6/230; 95% CI 0.96-5.59).

### RFPN per components

In all populations, low haptoglobin was the most frequent cause of RFPN, ranging from 0.41% to 1.30%.

#### Blood donors (P1)

Among the five (0.52%) tests with RFPN (Table 1), four (0.42%) were related to low haptoglobin and one to low A2M.

#### General population (P2)

Among the 38 (0.51%) tests with RFPN, 31 (0.41%) were related to low haptoglobin, five to high ApoA1, two to high GGT, one to low ApoA1 and one to high bilirubin with proven Gilbert's syndrome.

#### Worldwide population (P3) (Table 2)

**Table 2:** Prevalence of FibroTest with high-risk profile of false positive/negative (RFPN^1^)

Parameters	All (P3) n = 345,695		Reference center (P4) n = 24,872	
	**Lower limit and** ** RFPN n (%)**	**Upper limit and** ** RFPN n (%)**	**Lower limit and** ** RFPN n (%)**	**Upper limit and** ** RFPN n (%)**
**Haptoglobin g/L**	1590 (0.46%)^3^	0 (0%)^2^	324 (1.30%)	0 (0%)
**Apolipoprotein A1 g/L**	118 (0.03%)^3^	732 (0.21%)^2^	25 (0.10%)	57 (0.23%)

**Alpha-2 macroglobulin g/L**	419 (0.12%)^2^	427 (0.12%)^3^	54 (0.22%)	24 (0.10%)
**GGT IU/L**	0 (0%)	78 (0.02%)^3^	0 (0%)	8 (0.03%)
**Total Bilirubin, μmol/L**	0 (0%)	6 (0.001%)^3^	0 (0%)	0 (0%)

^1^Defined as a change > 0.30 in Fibrotest (at least 1.5 fibrosis METAVIR stage) when switching to the median value of the given parameter. Overall at least one parameter out of limits with significant impact was observed in 3349 cases (0.97%).

^2 ^Risk of false negative

^3 ^Risk of false positive

Among the 3,349 (0.97%) tests with RFPN, the most frequent were false positives due to low haptoglobin (0.46%), false negatives due to high ApoA1 (0.21%), false positives due to high A2M (0.12%), and false negatives due to low A2M (0.12%). The prevalence of other RFPN profiles (low apoA1, high GGT and high bilirubin) all together was 0.05%. No RFPN were related to high haptoglobin.

#### Reference centre patients (P4) (Table 2)

Among the 491 (1.97%) tests with RFPN, the most frequent were due to low haptoglobin (1.30%), and the others were similar to P3: high ApoA (0.23%), low A2M (0.22%), low ApoA1 (0.10%), high A2M (0.10%), high GGT (0.03%), and none for bilirubin.

### Factors associated with RFPN

#### General population (P2)

Sub-Saharan origin was the only factor associated with false positive due to low haptoglobin in multivariate analysis (OR = 8.0; 95% CI 3.2-20.0; P < 0.0001).

#### Worldwide patients (P3)

Factors associated in multivariate analysis with RFPN in the worldwide population (P3) are described in Table 3.

**Table 3:** Factors associated in multivariate analysis with RFPN in the worldwide population (P3)

	Low haptoglobin	Low ApoA1	High ApoA1	Low A2M	High A2M
**Risk for FibroTest**	**False Positive**	**False Positive**	**False Negative**	**False Negative**	**False Positive**

**Number of patients**	**1,590**	**118**	**732**	**419**	**427**

**Range**	**0.01-0.08 g/L**	**0.10-0.41 g/L**	**2.51-6.97 g/L**	**0.10-0.80 g/L**	**5.90-9.68 g/L**

					

**Factor (n)**	**Odds Ratio (95% CI) P value^1^**				
***Analytical factor***					
Last 3 years of test	0.76 (0.68-0.84) P < 10^-5^	NS	NS	NS	NS
> 10,000 tests	0.44 ((0.39-0.50) P < 10^-5^	NS	0.48 ((0.39-0.50) P < 10^-5^	NS	NS
***Non analytical factor***					
Residency					
Western Europe	NS	NS	NS	NS	NS
North Africa	NS	NS	NS	NS	NS
North America	0.05 (0.01-0.20) P = 0.00005	NS	NS	NS	NS
Eastern Europe	NS	NS	NS	22.44 (5.55-90.85) P < 10^-4^	NS
HIV center	3.97 (2.88-5.48) P < 10^-5^	NS	NS	NS	NS
Age > 50 years	0.63 (0.57-0.70) P < 10^-4^	NS	1.86 (1.60-1.86) P < 10^-5^	NS	1.56 (1.29-1.90) P = 10^-4^
Male gender	1.30 (1.18-1.45) P < 10^-4^	NS	0.34 (0.29-0.40) P < 10^-5^	2.6 (2.07-3.28) P < 10^-5^	NS
Reference center	5.35 (4.67-6.13) P < 10^-5^	2.61 (1.66-4.13) P = 0.00003	1.84 (1.39-2.45) P < 10^-5^	1.83 (1.36-2.47) P = 0.00008	NS

^1 ^Only odds ratio with P value lower or equal to 0.0001 were considered significant

##### Low A2M (Table 3 and Additional file 3, Table S5)

There was more risk of false negative in Eastern Europe residents.

##### High A2M (Table 3 and Additional file 3, Table S5)

There was more risk of false positives in Eastern Europe residents.

##### High Apolipoprotein A1 (Table 3 and Additional file 3, Table S6)

Both analytical and non-analytical factors were associated with a risk of false negative due to high ApoA1 in multivariate analysis. There was less risk in subjects assessed in laboratories with more experience, having performed over 10,000 FT-AT tests. There was more risk in subjects from the reference centre.

##### Low ApoA1 (Table 3 and additional file 3, Table S6)

Both analytical and non-analytical factors were associated with a risk of false positive due to low ApoA1 in multivariate analysis. There was more risk in laboratories with more experience (over 10,000 FT-ATs) and in the reference centre.

##### High GGT (additional file 3, Table S7), or high bilirubin

No risk factors were identified for false positive due to high GGT or high bilirubin.

##### Low haptoglobin (Table 3 and additional file 3, Table S7)

Both analytical and non-analytical factors were associated with the risk of false positive due to low haptoglobin in multivariate analysis. There was less risk in subjects assessed during the last 3 years of testing in laboratories with a high level of experience (more than 10,000 FT-AT), and in residents of Western Europe, North Africa and North America. There was more risk in HIV co-infected patients and in those from the reference centre.

##### Identification of analytical errors

The high rate of RFPN in Eastern Europe residents could be traced to one lab (#1517). This laboratory had a rise in RFPN prevalence from 1/65 (1.54%) in 2005, 12/166 (6.74%) in 2006 and 119/650 (18.31%) in 2007. The cause identified was an improper calibration that mistakenly settled the starting-point of the A2M calibration curve. After correction, the RFPN significantly decreased compared to previous years (P < 0.001): 58/1340 (4.33%) in 2008 and 26/1295 (2.01%) in 2009.

#### Reference centre patients (P4) (Table 4)

**Table 4:** Review of high risk FibroTest using charts in the reference center (P4)

Components of FibroTest with high risk profile	Number of Tests Identified	Reviewed (Traceability)^1^	Correctly classified		Indeterminate	Incorrectly classified	
			**True Positive**	**True Negative**		**False positive**	**False negative**
**Haptoglobin**							
< = 0.08 g/L	324	214 (66%)	58 (27%)	0 (0%)	42 (20%)	114 (53%)^2^	0 (0%)
> = 3.20 g/L	0	0 (100%)	0	0	0	0	0
**Apolipoprotein A1**							

< = 0.56 g/L	25	17 (68%)	15 (88%)	0	0 (0%)	2 (12%)^3^	0 (0%)

> = 2.50 g/L	57	38 (67%)	1 (3%)	30 (79%)	4 (10%)	0 (0%)	3 (8%)^4^

**Alpha-2 macroglobulin**							
< = 0.80 g/L	54	32 (59%)	3 (10%)	24 (75%)	0 (0%)	0 (0%)	5 (15%)^5^
> = 5.90 g/L	24	24 (100%)	22 (92%)	0	2 (8%)	0 (0%)	0 (0%)
**GGT IU/L > = 1140 IU/L**	8	6 (75%)	1 (16%)	1 (16%)	2 (33%)	2 (33%)^6^	0 (0%)
**Total Bilirubin, μmol/L > = 50**	0	0 (100%)	0	0	0	0	0
**Total**	491	331 (67%)	100 (30%)	55 (17%)	50 (14%)	118 (36%)	8 (2%)

^1 ^Inpatients with detailed charts reviewed by 3 experts. (Supplement file S3)

When biopsy was performed (5 years apart) it was taken as a reference; when no biopsy had been performed but an LSM was interpretable it was taken as a reference (advanced fibrosis if greater than 7.1 kPa); when oesophageal varices or ascites were present it was taken as advanced fibrosis; for low hapto, if no reference and a cause of hemolysis was identified FT > = 0.48 was considered false positive. When no reference was present without a clear cause of component error (such as hemolysis for hapto or severe undernutrition for A2M and apoA1, the case was stated to be indeterminate.

^2 ^Low haptoglobin, already known causes: hemolysis with patent cause: cardiac prosthesis (n = 25), drepanocytosis (n = 13), ribavirin (n = 8), thalassemia (n = 2), autoimmune (n = 2); anahaptoglobinemia (n = 1). Two new possible causes were HIV co-infection (n = 14) and splenectomy (n = 4)

^3 ^Low ApoA1 already known cause: severe undernutrition (n = 2 total serum proteins < = 50 g/L)

^4 ^High ApoA1 suspected analytical error or an unknown transient factor as in 2 cases, 2 repeated ApoA1 assays were in normal range

^5 ^Low A2M already known causes: large ascites (n = 3), severe undernutrition (n = 1 total serum proteins < = 50 g/L); a new possible cause was macrophage activation syndrome (n = 1)

^6 ^High GGT already known cause: chronic pancreatitis (n = 2)

Out of the 491 RFPN identified in the P4 population, 331 (67%) charts were prospectively reviewed (range 59-100%). Of these, 118 (36%) were proven false positives of FT, due to 1) low haptoglobin in 114 (mostly hemolysis) (Table 4 and additional file 4, Table S8); 2) high GGT in two (chronic pancreatitis) (Table 4 and additional file 5, Table S9); and 3) low ApoA1 in two (severe undernutrition) (Table 4 and additional file 5, Table S10). No specific cause of high A2M was found (additional file 5, Table S11). There were eight proven false negatives of FT: 5 due to low A2M (large ascites or severe undernutrition) (additional file 5, Table S12), and three due to high ApoA1 (additional file 5, Table S13). No specific cause of the ApoA1 increase was found, but two repeated assessments in two patients found a normal value, suggesting either an unknown transient factor or an analytical error for the first assay.

In 41/2602 (1.58%) HIV positive patients with haptoglobin RFPN, 14 (0.54%) were proven false positives, 18 proven true positives (0.69%) and in the remaining 9 (0.35%), the attributability of error was indeterminate.

Biopsies was the reference in 38 cases for suspected RPFN, including 5 for a FibroTest value stage F1 or F2 and an interval greater than one year between FibroTest and biopsy. LSM was the reference in 15 cases.

## Discussion

The present study assesses the applicability rate of FT-AT, using the definition of high-risk profiles of false positive/negative induced by each component. The prevalence of the most frequent proven causes of high-risk profiles was assessed, and new causes of high-risk profiles were identified. This study aim was to better define the applicability of such composite biomarkers. This study was not designed for the assessment of the classical false positive/false negative rate. Analysis of discordances between imperfect reference tests should be performed only among applicable results, and this specific topic has been discussed elsewhere [9]. We acknowledge that there is no ideal rule for the attribution of the cause of failure. That is why we use the simplest model one, that is a two classes discordances model, for the diagnosis of advanced fibrosis versus non advanced fibrosis with the predetermined standard threshold for FibroTest, biopsy and LSM. Due to the slow fibrosis progression rate, the risk of large variability is small in a two classes model (discordant patients were classified in a two classes model: F0F1 vs F2F34). Even for a rapid fibrosis progressor untreated (0.20 METAVIR stage per year), the mean change in 5 years is 1 stage. Therefore only patients in those patients stage F1 or in F2 patients sustained responders with a rapid regression, there was a risk of discordance due to an interval of 5 years or greater."

### Applicability

One criteria of efficiency of a diagnostic test is its applicability, defined as no test failure and reliable results. In the present study there was no FT failure and the applicability rate (reliable results) ranged from 99.49% in the general population to 98.03% among patients of the tertiary care reference centre. In comparison, the applicability of another validated marker of fibrosis, elastography, was much lower, with an 81.9% applicability rate assessed in 13,369 examinations, including 3.1% failure and 15.8% unreliable results [26].

### High-risk factors

The usual causes of RFPN were observed [4], but 3 new items of information were obtained. The RFPN prevalence in the general population, as well as their risk factors, was estimated, and in the reference centre the prevalence of proven specific causes was assessed.

#### Haptoglobin

Despite a limited rate (0.46%), low haptoglobin was clearly the most frequent cause of false positives. A significant independent risk (OR = 3.6) of haptoglobin false positive in patients with HIV was observed. The most frequent proven causes of low haptoglobin with RFPN were hemolysis due to cardiac prosthesis 25/114 (22%) and association with hemoglobin disease in 15/114 (13%), and HIV coinfection in 14/114 (12%).

A very low haptoglobin level had already been observed prospectively in 249 consecutive samples from HIV-infected subjects without any known cause of hemolysis [27] and was significantly associated with nucleoside analogues treatment. The accuracy of FT for the diagnosis of fibrosis remained highly significant in HIV patients coinfected with HCV [28] or HBV [29] and was similar to non-HIV patients. This is explained in the present study, as this profile rate which induced a significant change (more than one METAVIR fibrosis stage) is rare (1.58%), with only 0.54% proven false positives.

Only one case of anhaptoglobinemia was identified with RFPN. FT has not been prescribed widely in West African countries, a region which may have a higher prevalence of anahaptoglobulinemia. However a study in Burkina Faso did not observe such cases in a validation study of FT in patients with chronic hepatitis B [30].

Splenectomy was observed in four cases without other overt causes; we did not find any cases or rationale in the literature directly linking splenectomy and haptoglobin levels. The hypothesis could be that of a confounding disease (hemolytic anemia treated by splenectomy), or the consequences of splenectomy on red blood cell aging and destruction [31]. Therefore RFPN can not be directly attributed to splenectomy at this time. However this should be mentioned in the precautions of use.

We did not observe any cases of RFPN due to high haptoglobin, even in the tertiary care reference center. This is reassuring for patients with associated inflammatory disease and confirms the high applicability rate of FT already observed in patients with cryoglobulinemia and vasculitis [32].

#### ApoA1

High ApoA1 was the most frequent cause (0.21%) of false negative risk and was associated with the tertiary care reference center. The direct causality was uncertain. Among the three cases that indeed had a false negative result since advanced fibrosis was proven, the repeated apoA1 returned to "normal" values in two cases with true positives of repeated FT, suggesting a transient unknown factor or an analytical error. Among transient factors, a dietary cause was not identified, and we had already observed previously that there were no differences between fasting and non-fasting results [33].

Low ApoA1 was rarely (0.03%) associated with false positive risk. The only factor that was identified as being associated with this profile was the tertiary care reference center. Most of these patients had severe undernutrition with serum total protein concentrations lower than 50 g/L.

#### Alpha 2 macroglobulin

The reference center was associated both with false positive and false negative A2M RFPN results. The causes of low A2M proven false negatives were patients with large ascites or severe undernutrition with total proteins lower than 50 g/L. One new cause was a proven macrophage activation syndrome with a very high total protein count (112 g/L). The rationale could be an increase of IFN gamma [34], which down-regulates the A2M-activated receptor [35].

There were no cases of high A2M with false positive RFPN which were proven to be true false positives. Therefore it is possible that this profile could be an excessive security warning.

#### GGT

A very low prevalence of elevated GGT RFPN (0.02%) was observed. In the reference center, the only proven causes identified were two cases with chronic pancreatitis, an already well known cause of FT false positive.

#### Total bilirubin

The prevalence of bilirubin RFPN was very low, 0.001 in the worldwide database and none in the reference center. Only one case of Gilbert's syndrome was associated with a proven false positive among the general population. Overt Gilbert's syndrome has a prevalence of around 4%, but in the usual range of bilirubin levels, there was no significant impact on FT presumed fibrosis stage. These global results are reassuring, with a likely reliable physician selection for the exclusion of extra-hepatic cholestatic diseases.

#### Overall recommendations for precautions of use

As a comparison with the first HAS recommendations [3], this study estimated the prevalence of the previously identified intercurrent illnesses acting as confounding factors when interpreting test results: 0.46% for hemolysis, and less than 0.001% for Gilbert's syndrome. It was reassuring that there were no cases of acute inflammation identified through extreme values of haptoglobin or A2M. Despite a still high applicability rate, greater than 97%, the following at-risk populations must be mentioned: HIV infected patients and those of sub-Saharan origin. The following rare but proven causes must be added: severe undernutrition, pancreatitis, and macrophage activation syndrome. In addition, though it is not proven, splenectomy should be mentioned.

## Conclusion

This type of study should improve the benefit-risk assessment of non-invasive strategies in the forthcoming new standards of care of patients with chronic liver diseases [36]. Such studies must be performed in suspected high-risk populations as well as in apparently healthy volunteers which are representative of the general population. As for the approved drugs' labeling, the new diagnostic tests should describe the proven and suspected risk factors of false positive and negative with estimates of the prevalence of very high risk profiles, defining not applicable results.

## Competing interests

Thierry Poynard is the inventor of patented FibroTest and has a capital interest in Biopredictive the company marketing these tests. The patent belong to the French Public Organization "Assistance Publique Hopitaux de Paris". Mona Munteanu, Olivier Deckmyn, Yen Ngo, Fabienne Drane and Jean Marie Castille are full employee of Biopredictive.

## Authors' contributions

TP conceived of the study, write the article, performed the statistical analyses and participated in its design and coordination; MM and YN participated in the data management and statistical analyses; OD, FD and JMC participated in the data management; DM, CH and FIB participated in the biochemical assessments and the false positive/negative discussion; VR drafted the manuscript. All authors read and approved the final manuscript.

## Pre-publication history

The pre-publication history for this paper can be accessed here:

http://www.biomedcentral.com/1471-230X/11/39/prepub

## Supplementary Material

Additional file 1 **Publications concerning FibroTest**. Comprehensive list of publications concerning FibroTest performance. Click here for file

Additional file 2 **Prevalence of samples out of the initial reference range limits (P1, P2, P3)**. Table S1: Initial reference range for the parameters of FibroTest and ActiTest: Derived from first population definitions (Imbert-Bismut et al Lancet 2001, Myers HBV J Hep, Naveau ALD CGH and Ratziu NAFLD BMC Gastro). Table S2: Normal ranges in apparently healthy volunteers. Blood donors (P1). Distribution using initial references ranges in HCV, HBV, ALD and NAFLD. Table S3: Normal ranges in apparently healthy volunteers. General population P2, FibroTest components performed in the reference center. Table S4: Prevalence of samples out of the initial reference range limits, among global population (P3) Click here for file

Additional file 3 **Factors associated with high risk profile in patients' global population (P3)**. Tables S5: Alpha2 macroglobulin. Table S6: Apolipoprotein A1. Table S7: High haptoglobin and High GGT Click here for file

Additional file 4 **Patients details among patients of the reference tertiary care center (P4)**. Click here for file

Additional file 5 **Patients details among patients of the reference tertiary care center (P4) other than low haptoglobin**. Table S8: Low haptoglobin (< = 0.08 g/L) among 214 inpatients of reference center (P4). Table S9: High GGT > = 1140. Table S10: Low Apoa1 < = 0.56. Table S11: High A2M > = 5.90. Table S12: Low A2M (< = 0.80 g/L). Table S13: High Apoa1 > = 2.50. Click here for file
