# Supplementary material for: Applicability and precautions of use of liver injury biomarker FibroTest. A reappraisal at 7 years of age
Source: BMC Gastroenterol. 2011 Apr 14;11:39. doi: 10.1186/1471-230X-11-39 (PMC3097002; doi:10.1186/1471-230X-11-39)
Supplement: Additional file 1 — Publications concerning FibroTest. Comprehensive list of publications concerning FibroTest performance. [file 1471-230X-11-39-S1.DOCX]

**Additional files**

**Additional File 1: Comprehensive list of 157 publications concerning FibroTest's performance**

**Comprehensive list of publications concerning FibroTest performance (2001-2010)**

1. Imbert-Bismut F, Ratziu V, Pieroni L, Charlotte F, Benhamou Y, Poynard T. Biochemical markers of liver fibrosis in patients with hepatitis C virus infection: a prospective study. Lancet 2001; 357:1069-75.
2. Poynard T, Imbert-Bismut F, Ratziu V, Chevret S, Jardel C, Moussalli J, Messous D, Degos F. Biochemical markers of liver fibrosis in patients infected by Hepatitis C Virus: Longitudinal validation in a randomized trial. J Viral Hepatitis 2002; 9:128-133.
3. Myers RP, Ratziu V, Charlotte F, Imbert-Bismut F, Poynard T. Biochemical markers of liver fibrosis: a comparison with historical features in patients with chronic hepatitis C. Am J Gastroenterol 2002;97;2419-25.
4. Halfon P, Imbert-Bismut F, Messous D, Antoniotti G, Benchetrit D, Cart-Lamy P, Delaporte G, Doutheau D, Klump T, Sala M, Thibaud D, Trepo E, Robert P. Myers RP, Poynard T. A prospective assessment of the inter-laboratory variability of biochemical markers of fibrosis (FibroTest) and activity (ActiTest) in patients with chronic liver disease. Comparative Hepatology 2002; 2:3-7.
5. Myers RP, de Torres M, Imbert-Bismut F, Ratziu V, Charlotte F, Poynard T. Biochemical markers of fibrosis in patients with chronic hepatitis C: a comparison with prothrombin time, platelet count and the age-platelet index. Dig Dis Sci 2003;48:146-153.
6. Rossi E, Adams L, Prins A, Bulsara M, De Boer B, Garas G, MacQuillan G, Speers D, Jeffrey G. Validation of the FibroTest Biochemical Markers Score in Assessing Liver Fibrosis in Hepatitis C Patients. Clin Chem. 2003;49:450-454.
7. Poynard T, Imbert-Bismut F, Ratziu V, Myers RP, Di Martino V, Thabut D, Moussalli J, Benhamou Y. Fibrotest even better than liver biopsy ? Clin Chem 2003. Electronic letter http://www.clinchem.org/cgi/eletters/49/3/450. Response: (21 March 2003)
8. Myers RP, Benhamou Y, Imbert-Bismut F, Thibault V, Bochet M, Charlotte F, Ratziu V, Bricaire F, Katlama C, Poynard T. Serum biochemical markers accurately predict liver fibrosis in HIV and hepatitis C virus-coinfected patients. AIDS 2003;17:721-725.
9. Poynard T. Cost effectiveness of pegylated interferon alpha 2b and ribavirin combination in chronic hepatitis C. Gut. 2003;52:1532 (letter).
10. Thabut D, Simon M, Myers RP, Messous D, Thibaut V, Imbert-Bismut F, Poynard T. Noninvasive prediction of fibrosis in patients with chronic hepatitis C. Hepatology 2003; 37:1220-1. (letter)
11. Poynard T, McHutchison J, Manns M, Myers RP, Albrecht J. Biochemical surrogate markers of liver fibrosis and activity in a randomized trial of peginterferon alfa-2b and ribavirin. Hepatology. 2003;38:481-492.
12. Myers RP, Tainturier MH, Ratziu V, Piton A, Thibault V, Imbert-Bismut F, Messous D, Charlotte F, Di Martino V, Benhamou Y, Poynard T. Prediction of liver histological lesions with biochemical markers in patients with chronic hepatitis B. J Hepatol. 2003;39:222-30.
13. Le Calvez S, Thabut D, Messous D, Munteanu M, Ratziu V, Imbert-Bismut F, Poynard T. Fibrotest has higher predictive values than APRI for fibrosis diagnosis in patients with chronic hepatitis C. Hepatology 2004;39:862-3. (letter)
14. Imbert-Bismut F, Messous D, Thibaut V, Myers RB, Piton A, Thabut D, Devers L, Hainque B, Mercadier A, Poynard T. Intralaboratory analytical variability of biochemical markers of fibrosis (Fibrotest) and activity (Actitest) and reference ranges in healthy blood donors. Clin Chem Lab Med 2004;42:323-333.
15. Callewaert N, Van Vlierberghe H, Van Hecke A, Laroy W, Delanghe J, Contreras R. Noninvasive diagnosis of liver cirrhosis using DNA sequencer-based total serum protein glycomics. Nature Med 2004;10;429-34.
16. Poynard T, Imbert-Bismut F, Ratziu V. Serum markers of liver fibrosis. Hepatology Rev 2004;1:25-33.
17. Poynard T, Munteanu M, Imbert-Bismut F, Charlotte F, Thabut D, Le Calvez S, Messous D, Thibault V, Benhamou Y, Moussalli J, Ratziu V. Prospective Analysis of Discordant Results between Biochemical Markers and Biopsy in Patients with Chronic Hepatitis C. Clin Chem. 2004;50:1344-1355.
18. Munteanu M, Messous D, Thabut D, Imbert-Bismut F, Jouys M, Massard J, Piton A, Bonyhay L, Ratziu V, Hainque B, Poynard T. Intra-individual fasting versus postprandial variation of biochemical markers of liver fibrosis (FibroTest) and activity (ActiTest). Comp Hepatol 2004;3,3.
19. Poynard T, Imbert-Bismut F, Munteanu M, Messous D, Myers RP, Thabut D, Ratziu V, Mercadier A, Benhamou Y, Hainque B. Overview of the diagnostic value of biochemical markers of liver fibrosis (FibroTest, HCV-Fibrosure) and necrosis (ActiTest) in patients with chronic hepatitis C. Comp Hepatol 2004;3:8.
20. Ferard G, Imbert-Bismut F, Messous D, Piton A, Ueda S, Poynard T, Lessinger JM. A reference material for traceability of aspartate aminotransferase (AST) results. Clin Chem Lab Med. 2005;43:549-53.
21. Naveau S, Raynard B, Ratziu V, Abella A, Imbert-Bismut F, Messous D, Beuzen F, Capron F, Thabut D, Munteanu M, Chaput JC, Poynard T. Biomarkers for the prediction of liver fibrosis in patients with chronic alcoholic liver disease. Clin Gastroenterol Hepatol 2005;3:167-74.
22. Castéra L, Vergniol J, Foucher J, Brigitte Le Bail B, Chanteloup E, Haaser M, Darriet M, Couzigou P, de Lédinghen V. Prospective comparison of transient elastography, Fibrotest, APRI and liver biopsy for the assessment of fibrosis in chronic hepatitis C. Gastroenterology 2005;128:343-50.
23. Poynard T, Imbert-Bismut F, Munteanu M, Ratziu V. FibroTest-FibroSURE: towards a universal biomarker of liver fibrosis? Expert Rev Mol Diagn 2005;5:15-21.
24. Poynard T, Zoulim F, Ratziu V, Degos F, Imbert-Bismut F, Deny P, Landais P, El Hasnaoui A, Slama A, Blin P, Thibault V, Parvaz P, Munteanu M, Trepo C. Longitudinal assessment of histology surrogate markers (Fibrotest-Actitest) during lamivudine therapy in patients with chronic hepatitis B infection. Am J Gast 2005;100:1970-80.
25. Imbert-Bismut F, Messous D, Raoult A, Poynard T, Bertrand JJ, Marie PA, Louis V, Audy C, Thouy JM, Hainque B, Piton A. Results transferability on RXL, ARX, X-Pand, BN2 (Dade Behring) and modular DP (Roche Diagnostics) analysers: application to component assays of fibrotest and Actitest Ann Biol Clin (Paris). 2005;63:305-13.
26. Piton A, Messous D, Imbert-Bismut F, Berges J, Munteanu M, Poynard T, Hainque B. Alpha 2 macroglobulin immunoturbidimetric assays (DakoCytomation reagents) on Roche Diagnostic analysers (Modular P, Cobas Integra). Application to FibroTest-ActiTest. Ann Biol Clin (Paris). 2005;63:385-95.
27. Poynard T, Ratziu V, Benhamou Y, Thabut D, Moussalli J. Biomarkers as a first-line estimate of injury in chronic liver diseases: time for a moratorium on liver biopsy? Gastroenterology. 2005;128:1146-8.
28. Poynard T, Ratziu V, Imbert-Bismut F, Munteanu M.   Fibrosis biomarkers are not only a formula. Clin Chem 2005. http://www.clinchem.org/cgi/eletters/51/10/1867. (6 September 2005). Electronic letter.
29. Cales P, Oberti F, Michalak S, Hubert-Fouchard I, Rousselet MC, Konate A, Gallois Y, Ternisien C, Chevailler A, Lunel F. A novel panel of blood markers to assess the degree of liver fibrosis. Hepatology. 2005;42:1373-81.
30. Colletta C, Smirne C, Fabris C, Toniutto P, Rapetti R, Minisini R, Pirisi M. Value of two noninvasive methods to detect progression of fibrosis among HCV carriers with normal aminotransferases. Hepatology. 2005;42:838-45.
31. Poynard T, Ratziu V, Naveau S, Thabut D, Charlotte F, Messous D, Capron D, Abella A, Massard J, Ngo Y, Munteanu M, Mercadier A, Manns M, Albrecht J. The diagnostic value of biomarkers (SteatoTest) for the prediction of liver steatosis. Comp Hepatol. 2005;4:10
32. Varaut A, Fontaine H, Serpaggi J, Verkarre V, Vallet-Pichard A, Nalpas B, Imbert Bismuth F, Lebray P, Pol S. Diagnostic accuracy of the fibrotest in hemodialysis and renal transplant patients with chronic hepatitis C virus. Transplantation. 2005;80:1550-5.
33. Rosenthal-Allieri MA, Peritore ML, Tran A, Halfon P, Benzaken S, Bernard A. Analytical variability of the Fibrotest proteins. Clin Biochem. 2005;38:473-8.
34. D’Arondel C, Munteanu M, Moussalli J, Thibault V, Naveau S, Simon A, Messous D, Morra R, Blot C, Poynard T. A prospective assessment of an “a la carte" regimen of PEG Interferon alfa 2b and ribavirin combination in patients with chronic hepatitis C using biochemical markers. J Vir Hep 2006;13:182-9.
35. Ratziu V, Massard J, Charlotte F, Messous D, Imbert-Bismut F, Bonyhay L, Tahiri M, Munteanu M, Thabut D, Cadranel JF, Le Bail B, De Ledinghen V, Poynard T, the LIDO Study Group and the CYTOL Study Group. Diagnostic value of biochemical markers (FibroTest-FibroSURE) for the prediction of liver fibrosis in patients with non-alcoholic fatty liver disease. BMC Gastroenterology 2006, 6:6.
36. Castera L, Foucher J, Bertet J, Couzigou P, de Ledinghen V. FibroScan and FibroTest to assess liver fibrosis in HCV with normal aminotransferases. Hepatology. 2006;43:373-4. (Letter)
37. Poynard T, Munteanu M, Ngo Y, Torres M, Benhamou Y, Thabut D, Ratziu V. Diagnostic value of FibroTest with normal serum aminotransferases. Hepatology. 2006;43:374-5. (Letter).
38. Halfon P, Bourliere M, Deydier R, Portal. I, Renou R, Bertrand J, Trana A, Rosenthal A, Rotily M, Sattonet A. Independent prospective multicenter validation of biochemical markers (Fibrotest-Actitest) for the prediction of liver fibrosis and activity in patients with chronic hepatitis C. Am J Gastro 2006;101:547–555.
39. Ferard G, Piton A, Messous D, Imbert-Bismut F, Frairi A, Poynard T, Lessinger JM. Intermethod calibration of alanine aminotransferase (ALT) and gamma-glutamyltransferase (GGT) results: application to Fibrotest and Actitest scores. Clin Chem Lab Med. 2006;44:400-6.
40. Thabut D, Trabut JB, Massard J, Rudler M, Muntenau M, Messous D, Poynard T. Non-invasive diagnosis of large oesophageal varices with FibroTest in patients with cirrhosis: a preliminary retrospective study. Liver Int. 2006;26:271-8.
41. Thabut D, Naveau S, Charlotte F, Massard J, Ratziu V, Imbert-Bismut F,Cazals-Hatem D, Abella A, Messous D, Beuzen F, Munteanu M, Taieb J, Moreau R, Lebrec D, Poynard T. The diagnostic value of biomarkers (AshTest) for the prediction of alcoholic steato-hepatitis in patients with chronic alcoholic liver disease. J Hepatol. 2006;44:1175-85.
42. Sebastiani G, Vario A, Guido M, Noventa F, Plebani M, Pistis R, Ferrari A, Alberti A. Stepwise combination algorithms of non-invasive markers to diagnose significant fibrosis in chronic hepatitis C. J Hepatol. 2006;44:686-93.
43. Wilson LE, Torbenson M, Astemborski J, Faruki H, Spoler C, Rai R, Mehta S, Kirk GD, Nelson K, Afdhal N, Thomas DL. Progression of liver fibrosis among injection drug users with chronic hepatitis C. Hepatology. 2006;43:788-95.
44. Sène D , Limal N, Messous D, Ghillani-Dalbin P, Charlotte F, Halfon P, Thiollière JM, Piette JC, Imbert-Bismut F, Halfon P, Poynard T, Cacoub P. Biological markers of liver fibrosis and activity as non-invasive alternatives to liver biopsy in patients with chronic hepatitis C and associated mixed cryoglobulinemia vasculitis. Clin Biochem 2006;39:715-21.
45. Thabut D, Le Calvez S, Thibault V, Massard J, Munteanu M, Di Martino V, Ratziu V, Poynard T. Hepatitis C in 6,865 Patients 65 yr or Older: A severe and neglected curable disease? Am J Gastroenterol. 2006;101:1260-7.
46. Maor Y, Bashari D, Kenet G, Lubetsky A, Luboshitz J, Schapiro JM, Penaranda G, Bar-Meir S, Martinowitz U, Halfon P. Non-invasive biomarkers of liver fibrosis in haemophilia patients with hepatitis C: can you avoid liver biopsy? Haemophilia. 2006;12:372-9.
47. Ngo Y, Munteanu M, Messous D, Charlotte F, Imbert-Bismut F, Thabut D, Lebray P, Thibault V, Benhamou Y, Moussalli J, Ratziu V, Poynard T. A prospective analysis of the prognostic value of biomarkers (FibroTest) in patients with chronic hepatitis C. Clin Chem. 2006;52:1887-96.
48. Poynard T, Ratziu V, Charlotte F, Messous D, Munteanu M, Imbert Bismut F, Massard J, Bonyhay L, Tahiri M, Thabut D, Cadranel JF, Le Bail B, de Ledinghen V, Group L, Group C. Diagnostic value of biochemical markers (NashTest) for the prediction of NASH in patients with non-alcoholic fatty liver disease. BMC Gastroenterol. 2006;6:34.
49. Bourliere M, Penaranda G, Renou C, Botta-Fridlund D, Tran A, Portal I,Lecomte L, Castellani P, Rosenthal-Allieri MA, Gerolami R, Ouzan D, Deydier R, Degott C, Halfon P. Validation and comparison of indexes for fibrosis and cirrhosis prediction in chronic hepatitis C patients: proposal for a pragmatic approach classification without liver biopsies. J Viral Hepat. 2006;13:659-70.
50. Ratziu V, Giral P, Munteanu M, Messous D, Mercadier A, Bernard M, Morra R, Imbert-Bismut F, Bruckert E, Poynard T. Screening for liver disease using non-invasive biomarkers (FibroTest-SteatoTest-NashTest-FibroSURE) in patients with hyperlipidaemia. Aliment Pharmacol Ther. 2007;25:207-18.
51. Halfon P, Bacq Y, De Muret A, Penaranda G, Bourliere M, Ouzan D, Tran A, Botta D, Renou C, Brechot MC, Degott C, Paradis V. Comparison of test performance profile for blood tests of liver fibrosis in chronic hepatitis C. J Hepatol. 2007;46:395-402.
52. Poynard T, Halfon P, Castera L, Charlotte F, Bail BL, Munteanu M, Messous D, Ratziu V, Benhamou Y, Bourliere M, Ledinghen VD; the FIBROPACA group. Variability of the area under the receiver operating characteristic curves in the diagnostic evaluation of liver fibrosis markers: impact of biopsy length and fragmentation. Aliment Pharmacol Ther. 2007;25:733-739.
53. Sebastiani G, Vario A, Guido M, Alberti A. Sequential algorithms combining non-invasive markers and biopsy for the assessment of liver fibrosis in chronic hepatitis B. World J Gastroenterol. 2007;13:525-31.
54. Leroy V, Hilleret MN, Sturm N, Trocme C, Renversez JC, Faure P, Morel F, Zarski JP. Prospective comparison of six non-invasive scores for the diagnosis of liver fibrosis in chronic hepatitis C. J Hepatol. 2007 ;46 :775-82.
55. Grigorescu M, Rusu M, Neculoiu D, Radu C, Aerban A, Caþanao M, Grigorescu MD. The FibroTest value in discriminating between insignificant and significant fibrosis in chronic hepatitis C patients. The Romanian Experience Gastrointestin Liver Dis 2007; 16: 31-37l.
56. Berends MA, Snoek J, de Jong EM, Van Krieken JH, de Knegt RJ, van Oijen MG, van de Kerkhof PC, Drenth JP. Biochemical and biophysical assessment of MTX-induced liver fibrosis in psoriasis patients: Fibrotest predicts the presence and Fibroscan predicts the absence of significant liver fibrosis. Liver Int. 2007;27:639-45.
57. Vallet-Pichard A, Mallet V, Nalpas B, Verkarre V, Nalpas A, Dhalluin-Venier V, Fontaine H, Pol S. FIB-4: an inexpensive and accurate marker of fibrosis in HCV infection. Comparison with liver biopsy and Fibrotest. Hepatology. 2007;46:32-6.
58. Morra R, Munteanu M, Imbert-Bismut F, Messous D, Ratziu V, Poynard T. FibroMAX: towards a new universal biomarker of liver disease? Expert Rev Mol Diagn. 2007;7:481-90.
59. Poynard T, Halfon P, Castera L, Munteanu M, Imbert-Bismut F, Ratziu V, Benhamou Y, Bourliere M, de Ledinghen V, FibroPaca Group. Standardization of ROC curve areas for diagnostic evaluation of liver fibrosis markers based on prevalences of fibrosis stages. Clin Chem. 2007;53:1615-1622.
60. Thabut D, Imbert-Bismut F, Cazals-Hatem D, Messous D, Muntenau M, Valla DC, Moreau R, Poynard T, Lebrec D. Relationship between the Fibrotest and portal hypertension in patients with liver disease. Aliment Pharmacol Ther. 2007;26:359-368.
61. Morra R, Munteanu M , Bedossa P, Dargere D, Janneau JL, Paradis V, Ratziu V, Charlotte F, Thibault V, Imbert-Bismut F, Poynard T. Diagnostic value of serum protein profiling by SELDI-TOF ProteinChip compared with a biochemical marker, FibroTest, for the diagnosis of advanced fibrosis in patients with chronic hepatitis C. Aliment Pharmacol Ther. 2007; 26:847-58.
62. Coco B, Oliveri F, Maina AM, Ciccorossi P, Sacco R, Colombatto P, Bonino F, Brunetto MR. Transient elastography: a new surrogate marker of liver fibrosis influenced by major changes of transaminases. J Viral Hepat. 2007;14:360-9.
63. Morali G, Maor Y, Klar R, Braun M, Ben Ari Z, Bujanover Y, Zuckerman E, Boger S Halfon P. Fibrotest-Actitest: the biochemical marker of liver fibrosis – The Israeli Experience. Israeli Med Assoc J. 2007; 9:588-91.
64. Lewin M, Poujol-Robert A, Boelle PY, Wendum D, Lasnier E, Viallon M, GuechotJ, Hoeffel C, Arrive L, Tubiana JM, Poupon R. Diffusion-weighted magnetic resonance imaging for the assessment of fibrosis in chronic hepatitis C. Hepatology. 2007;46:658-65.
65. Poynard T, Morra R, Halfon P, Castera L, Ratziu V, Imbert-Bismut F, Naveau S, Thabut D, Lebrec D, Zoulim F, Bourliere M, Cacoub P, Messous D, Munteanu M, de Ledinghen V. Meta-analyses of Fibrotest diagnostic value in chronic liver disease BMC Gastroenterology 2007, 7:40.
66. Calès P, Veillon P, Konaté A, Mathieu E, Ternisien C, Chevailler A, Godon A, Gallois Y, Joubaud F, Hubert-Fouchard I, Oberti F, Réaud S, Hunault G, Mauriat F, Lunel-Fabiani F, Reproducibility of blood tests of liver fibrosis in clinical practice. Clin Biochem. 2008;41:10-18.
67. de Lédinghen V, Le Bail B, Rebouissoux L, Fournier C, Foucher J, Miette V,Castéra L, Sandrin L, Merrouche W, Lavrand F, Lamireau T. Liver stiffness measurement in children using FibroScan: feasibility study and comparison with Fibrotest, aspartate transaminase to platelets ratio index, and liver biopsy. J Pediatr Gastroenterol Nutr. 2007;45:443-50.
68. Maor Y, Calès P, Bashari D, Kenet G, Lubetsky A, Luboshitz J, Schapiro JM, Pénaranda G, Bar-Meir S, Martinowitz U, Halfon P. Improving estimation of liver fibrosis using combination and newer non-invasive biomarker scoring systems in hepatitis C-infected haemophilia patients. Haemophilia. 2007;13:722-9.
69. Shaheen AA, Wan AF, Myers RP. FibroTest and FibroScan for the prediction of hepatitis C-related fibrosis: a systematic review of diagnostic test accuracy. Am J Gastroenterol. 2007;102:2589-600.
70. Fontaine H, Petitprez K, Roudot-Thoraval F, Trinchet JC; Association francaise pour l'etude du foie; Club de reflexion des cabinets et groupes d'hepato-gastro-enterologue; College national des generalistes enseignants; Groupe francophone d'hepatologie gastro-enterologie et nutrition pediatriques; Societe francaise de biologie clinique; Societe francaise de radiologie; Societe nationale francaise de gastro-enterologie. Guidelines for the diagnosis of uncomplicated cirrhosis. Gastroenterol Clin Biol. 2007;31:504-9.
71. La Haute Autorite´ de Sante´ (HAS) in France—The HAS recommendations for the management of the chronic hepatitis C using non‐invasive biomarkers. http://www.has‐sante.fr/portail/display.jsp?id ¼c_476486 (Accessed August 2008).
72. Castera L, Denis J, Babany G, Roudot-Thoraval F. Evolving practices of non-invasive markers of liver fibrosis in patients with chronic hepatitis C in France: time for new guidelines?. J Hepatol. 2007;46:528-9.
73. Morra R, Lebray P, Ingiliz P, Ngo Y, Munteanu M, Ratziu V, Poynard T. FibroTest has better diagnostic and prognostic values than the aspartate aminotransferase-to-platelet ratio index in patients with chronic hepatitis C. Hepatology. 2008;47:353-4.
74. Adler M, Gulbis B, Moreno C, Evrard S, Verset G, Golstein P, Frotscher B, Nagy N, Thiry P. The predictive value of FIB-4 versus FibroTest, APRI, FibroIndex and Forns index to noninvasively estimate fibrosis in hepatitis C and nonhepatitis C liver diseases. Hepatology. 2008;47:762-3.
75. Sebastiani G, Vario A, Guido M, Alberti A. Performance of noninvasive markers for liver fibrosis is reduced in chronic hepatitis C with normal transaminases. Journal of Viral Hepatitis. 2008, 15, 212–8
76. Poynard T, Munteanu M, Ngo Y, Moussalli J, Lebray P, Thabut D, Benhamou Y, Ratziu V. FibroTest is effective in patients with normal transaminases, when accuracy is standardized on fibrosis stage prevalence. J Viral Hepat. 2008;15:472-3.
77. Cacoub P, Carrat F, Bédossa P, Lambert J, Pénaranda G, Perronne C, Pol S, Halfon P. Comparison of non-invasive liver fibrosis biomarkers in HIV/HCV co-infected patients: The Fibrovic study - ANRS HC02. J Hepatol. 2008;48:765-73.
78. Rosenthal-Allieri MA, Tran A, Halfon P, Imbert-Bismut F, Munteanu M, Messous D, Peritore ML, Poynard T, Bernard A. Optimal correlation between different instruments for Fibrotest-Actitest protein measurement in patients with chronic hepatitis C. Gastroenterol Clin Biol. 2007;31:815-21.
79. Friedrich-Rust M, Koch C, Rentzsch A, Sarrazin C, Schwarz P, Herrmann E, Lindinger A, Sarrazin U, Poynard T, Schäfers HJ, Zeuzem S, Abdul-Khaliq H. Noninvasive assessment of liver fibrosis in patients with Fontan circulation using transient elastography and biochemical fibrosis markers. J Thorac Cardiovasc Surg. 2008;135:560-7.
80. Shaheen AA, Myers RP. Systematic review and meta-analysis of the diagnostic accuracy of fibrosis marker panels in patients with HIV/hepatitis C coinfection. HIV Clin Trials. 2008;9:43-51.
81. Jacqueminet S, Lebray P, Morra R, Munteanu M, Devers L, Messous D, Bernard M, Hartemann-Heurtier A, Imbert-Bismut F, Ratziu V, Grimaldi A, Poynard T. Screening for liver fibrosis by using a noninvasive biomarker in patients with diabetes. Clin Gastroenterol Hepatol. 2008;6:828-31.
82. Bourliere M, Penaranda G, Ouzan D, Renou C, Botta-Fridlund D, Tran A, Rosenthal E, Wartelle-Bladou C, Delasalle P, Oules V, Portal I, Castellani P, Lecomte L, Rosenthal-Allieri MA, Halfon P. Optimized stepwise combination algorithms of non invasive liver fibrosis scores including Hepascore in HCV patients. Aliment Pharmacol Ther. 2008;28:458-465.
83. Calès P, de Ledinghen V, Halfon P, Bacq Y, Leroy V, Boursier J, Foucher J, Bourlière M, de Muret A, Sturm N, Hunault G, Oberti F. Evaluating the accuracy and increasing the reliable diagnosis rate of blood tests for liver fibrosis in chronic hepatitis C. Liver Int. 2008;28:1352-62.
84. Munteanu M, Imbert-Bismut F, Messous D, Morra R, Thabut D, Lebray P, Benhamou Y, Ratziu V, Poynard T. Reproducibility of non-invasive fibrosis biomarkers, FibroMeter and FibroTest, could be improved by respecting the analytical standardizations. Clin Biochem. 2008;41:1113-4
85. Ngo Y, Benhamou Y, Thibault V, Ingiliz P, Munteanu M, Lebray P, Thabut D, Morra R, Messous D, Charlotte F, Imbert-Bismut F, Rousselot-Bonnefont D, Moussalli J, Ratziu V, Poynard T. An accurate definition of the status of inactive hepatitis B virus carrier by a combination of biomarkers (Fibrotest-Actitest) and viral load. PlosOne 2008;3:e2573.
86. Imbert-Bismut F, Naveau S, Morra R, Munteanu M, Ratziu V, Abella A, Messous D, Thabut D, Benhamou Y, Poynard T. The diagnostic value of combining carbohydrate deficient transferin, fibrosis and steatosis biomarkers for the prediction of excessive alcohol consumption. Eur J Gastro Hepatol 2009;21:18-27.
87. Munteanu M, Ratziu V, Morra R, Messous D, Imbert-Bismut F, Poynard T. Noninvasive biomarkers for the screening of fibrosis, steatosis and steatohepatitis in patients with metabolic risk factors: FibroTest-FibroMax Experience. J Gastrointestin Liver Dis. 2008;17:187-91.
88. Poynard T, Morra R, Ingiliz P, Imbert‐Bismut F, Thabut D, Messous D, Muntenau M, Massard J, Benhamou Y, Ratziu V. Biomarkers of liver fibrosis. Adv Clin Chem 2008; 46: 131-152.
89. Zhao LR , Xu DJ, Lu ZH, Zhao H, Lang ZW, Wang GQ. Validation of FibroTest to diagnose liver fibrosis to patients with chronic hepatitis B. Chinese Journal of Practical Internal Medicine 2007; 27:1274-1277.
90. Naveau S, Gaudé G, Asnacios A, Agostini H, Abella A, Barri-Ova N, Dauvois B, Prévot S, Ngo Y, Munteanu M, Balian A, Njiké-Nakseu M, Perlemuter G, Poynard T. Diagnostic and prognostic values of non-invasive biomarkers of fibrosis in patients with alcoholic liver disease. Hepatology 2009; 49:97-105.
91. Poynard T, Ngo Y, Marcellin P, Hadziyannis S, Ratziu V, Benhamou Y. Impact of adefovir dipivoxil on liver fibrosis and activity assessed with biochemical markers (FibroTest-ActiTest) in patients infected by Hepatitis B Virus. J Vir Hep 2009; 16:203-13.
92. Patel K, Benhamou Y, Yoshida EM, Kaita KD, Zeuzem S, Torbenson M, Pulkstenis E, M. Subramanian M, McHutchison JG. An independent and prospective comparison of two commercial fibrosis marker panels (HCV FibroSURE and FIBROSpect II) during albinterferon alfa-2b combination therapy for chronic hepatitis C. J Vir Hep 2009; 16:178-86.
93. Poynard T, Ingiliz P, Elkrief L, Munteanu M, Lebray P, Morra R, Messous D, Imbert Bismut F, Roulot D, Benhamou Y, Thabut D, Ratziu V. Concordance in a world without a gold standard: A new non-invasive methodology for improving accuracy of fibrosis markers. PlosOne 2008. 3:e 3857.
94. Poynard T, Muntenau M, Morra R, Ngo Y, Imbert-Bismut F, Thabut D, Messous D, Massard J, Lebray P, Moussalli J, Benhamou Y, Ratziu V. Methodological aspects for the interpretation of liver fibrosis non-invasive biomarkers: a 2008 update. Gastroenterol Clin Biol 2008;32:8-21.
95. Halfon P, Munteanu M, Poynard T. FibroTest-ActiTest as a non-invasive marker of liver ﬁbrosis. Gastroenterol Clin Biol 2008;32:22-38.
96. Dolmazashvili E, Zhamutashvili M, Svanidze M, Nizharadze N, Abutidze A. Fibroscan and FibroTest/FibroMax to assess liver fibrosis/cirrhosis in patients with chronic HBV and HCV infection in Georgia. Georgian Med News. 2008;165:83-7. No pdf
97. Castéra L, Bail BL, Roudot-Thoraval F, Bernard PH, Foucher J, Merrouche W, Couzigou P, de Lédinghen V. Early detection in routine clinical practice of cirrhosis and oesophageal varices in chronic hepatitis C: Comparison of transient elastography (FibroScan) with standard laboratory tests and non-invasive scores. J Hepatol. 2009;50:59-68.
98. Boursier J, Bacq Y, Halfon P, Leroy V, de Ledinghen V, de Muret A, Bourlière M, Sturm N, Foucher J, Oberti F, Rousselet MC, Calès P. Improved diagnostic accuracy of blood tests for severe fibrosis and cirrhosis in chronic hepatitis C. Eur J Gastroenterol Hepatol. 2009;21:28-38.
99. Gui HL, Xie Q, Wang H, Zhou HJ, Cai W, An BY, Jiang S, Xu B, Lin ZM, Xu YM. FibroTest-ActiTest for predicting liver fibrosis and inflammatory activity in Chinese patients with chronic hepatitis B. Zhonghua Gan Zang Bing Za Zhi. 2008;16:897-901.
100. Nguyen-Khac E, Chatelain D, Tramier B, Decrombecque C, Robert B, Joly JP, Brevet M, Grignon P, Lion S, Le Page L, Dupas JL. Assessment of asymptomatic liver fibrosis in alcoholic patients using fibroscan: prospective comparison with seven non-invasive laboratory tests. Aliment Pharmacol Ther. 2008;28:1188-98.
101. Zelber-Sagi S, Nitzan-Kaluski D, Goldsmith R, Webb M, Zvibel I, Goldiner I, Blendis L, Halpern Z, Oren R. Role of leisure-time physical activity in nonalcoholic fatty liver disease: a population-based study. Hepatology. 2008;48:1791-8.
102. Alric L, Kamar N, Bonnet D, Danjoux M, Abravanel F, Lauwers-Cances V, Rostaing L. Comparison of liver stiffness, fibrotest and liver biopsy for assessment of liver fibrosis in kidney-transplant patients with chronic viral hepatitis. Transpl Int. 2009;22:568-73.
103. Salles N, Dussarat P, Foucher J, Villars S, de Lédinghen V. Non-invasive evaluation of liver fibrosis by transient elastography and biochemical markers in elderly inpatients. Gastroenterol Clin Biol. 2009;33:126-32.
104. Vanderschaeghe D, Laroy W, Sablon E, Halfon P, Van Hecke A, Delanghe J, Callewaert N. GlycoFibroTest is a highly performant liver fibrosis biomarker derived from DNA sequencer-based serum protein glycomics. Mol Cell Proteomics. 2009;8:986-94.
105. Vergniol J, Foucher J, Castéra L, Bernard PH, Tournan R, Terrebonne E,Chanteloup E, Merrouche W, Couzigou P, de Lédinghen V. Changes of non-invasive markers and FibroScan values during HCV treatment. J Viral Hepat. 2009;16:132-40.
106. Sebastiani G, Halfon P, Castera L, Pol S, Thomas DL, Mangia A, Marco VD, Pirisi M, Voiculescu M, Guido M, Bourliere M, Noventa F, Alberti A. SAFE biopsy: A validated method for large-scale staging of liver fibrosis in chronic hepatitis C. Hepatology. 2009:49:1821-7.
107. Lau-Corona D, Pineda LA, Aviles HH, Gutierrez-Reyes G, Farfan-Labonne BE, Nunez-Nateras R, Bonder A, Martinez-Garcia R, Corona-Lau C, Olivera-Martinez MA, Gutierrez-Ruiz MC, Robles-Diaz G, Kershenobich D. Effective use of FibroTest to generate decision trees in hepatitis C. World J Gastroenterol 2009;15:2617-22.
108. Poynard T, Benhamou Y, Thabut D, Ratziu V. Liver biopsy: the best standard...when everything else fails. J Hepatol. 2009 ;50:1267-8.
109. Poynard T, Morra R, Ingiliz P, Imbert-Bismut F, Thabut D, Messous D, Munteanu M, Massard J, Benhamou Y, Ratziu V. Assessment of liver fibrosis: noninvasive means. Saudi J Gastroenterol. 2008;14:163-73.
110. Fontanges T, Bailly F, Trepo E, Chevallier M, Maynard-Muet M, Nalet B, Beorchia S, Pillon D, Moindrot H, Froissart B, Slaoui M, Tinel X, Pradat P, Trepo C. Discordance between biochemical markers of liver activity and fibrosis (Actitest-Fibrotest) and liver biopsy in patients with chronic hepatitis C. Gastroenterol Clin Biol. 2008;32: 858-65.
111. Poynard T, Munteanu M, Ngo Y, Ratziu V. Appropriate evidence-based data overviews demonstrate the diagnostic and prognostic performances of FibroTest in patients with chronic hepatitis C. Aliment Pharmacol Ther. 2009;30:1183-5.
112. Anastasiou J, Alisa A, Virtue S, Portmann B, Murray-Lyon I, Williams R. Noninvasive markers of fibrosis and inflammation in clinical practice: prospective comparison with liver biopsy. Eur J Gastroenterol Hepatol. 2010;22:474-80.
113. Gressner OA, Beer N, Jodlowski A, Gressner AM. Impact of quality control accepted inter-laboratory variations on calculated Fibrotest/Actitest scores for the non-invasive biochemical assessment of liver fibrosis. Clin Chim Acta. 2009;409:90-5.
114. Friedrich-Rust M, Schwarz A, Ong M, Dries V, Schirmacher P, Herrmann E, Samaras P, Bojunga J, Bohle RM, Zeuzem S, Sarrazin C. Real-time tissue elastography versus FibroScan for noninvasive assessment of liver fibrosis in chronic liver disease. Ultraschall Med. 2009;30:478-84.
115. Hermeziu B, Messous D, Fabre M, Munteanu M, Baussan C, Bernard O, Poynard T, Jacquemin E. Evaluation of FibroTest-ActiTest in children with chronic hepatitis C virus infection. Gastroenterol Clin Biol. 2010;34:16-22.
116. Boursier J, Vergniol J, Sawadogo A, Dakka T, Michalak S, Gallois Y, Le Tallec V, Oberti F, Fouchard-Hubert I, Dib N, Rousselet MC, Konate A, Amrani N, de Ledinghen V, Cales P. The combination of a blood test and Fibroscan improves the non-invasive diagnosis of liver fibrosis. Liver Int. 2009;29:1507-15.
117. Rubio A, Monpoux F, Huguon E, Truchi R, Triolo V, Rosenthal-Allieri MA, Deville A, Rosenthal E, Boutte P, Tran A. Noninvasive procedures to evaluate liver involvement in HIV-1 vertically infected children. J Pediatr Gastroenterol Nutr. 2009;49:599-606.
118. Maor Y, Halfon P, Bashari D, Penaranda G, Morali G, Klar R, Bar-Meir S, Martinowitz U, Oren R. Fibrotest or Fibroscan for evaluation of liver fibrosis in haemophilia patients infected with hepatitis C. Haemophilia. 2009. No Pdf.
119. Ingiliz P, Chhay KP, Munteanu M, Lebray P, Ngo Y, Roulot D, Benhamou Y, Thabut D, Ratziu V, Poynard T. Applicability and variability of liver stiffness measurements according to probe position. World J Gastroenterol. 2009;15:3398-404.
120. Friedrich-Rust M, Muller C, Winckler A, Kriener S, Herrmann E, Holtmeier J, Poynard T, Vogl TJ, Zeuzem S, Hammerstingl R, Sarrazin C. Assessment of Liver Fibrosis and Steatosis in PBC With FibroScan, MRI, MR-spectroscopy, and Serum Markers. J Clin Gastroenterol. 2010;44:58-65.
121. Halfon P, Carrat F, Bédossa P, Lambert J, Penaranda G, Perronne C, Pol S, Cacoub P. Effect of antiviral treatment on serum markers of liver fibrosis in HIV-hepatitis C virus-coinfected patients: the Fibrovic 2 Study - ANRS HC02. Antivir Ther. 2009;14:211-9.
122. Bottero J, Lacombe K, Guéchot J, Serfaty L, Miailhes P, Bonnard P, Wendum D, Molina JM, Lascoux-Combe C, Girard PM. Performance of 11 biomarkers for liver fibrosis assessment in HIV/HBV co-infected patients. J Hepatol. 2009;50:1074-83.
123. Pavlov CS, Glushenkov DV, Konovalova ON, Ivashkin VT. The scope of clinical applications of noninvasive methods for the assessment of liver fibrosis results of orginial studies in a multi-field hospital. Klin Med (Mosk). 2009;87:40-5. No pdf
124. Castera L, Sebastiani G, Le Bail B, de Ledinghen V, Couzigou P, Alberti A. Prospective comparison of two algorithms combining non-invasive methods for staging liver fibrosis in chronic hepatitis C. J Hepatol 2010;52:191-8.
125. Gui HL, Gao CF, Wang H, Liu XE, Xie Q, Dewaele S, Wang L, Zhuang H, Contreras R, Libert C, Chen C. Altered serum N-glycomics in chronic hepatitis B patients. Liver Int. 2010;30:259-67.
126. Laharie D, Vergniol J, Bioulac-Sage P, Diris B, Poli J, Foucher J, Couzigou P, Drouillard J, de Ledinghen V. Usefulness of noninvasive tests in nodular regenerative hyperplasia of the liver. Eur J Gastroenterol Hepatol. 2010;22:487-93.
127. Munteanu M, Imbert-Bismut F, Housset C, Messous D, Fellahi S, Poynard T. Evaluation of FibroTest inter-laboratory variations requires standardization on analytical systems and should not be mixed with underpowered histological validation. Clin Chim Acta 2010;411:3-4.
128. Poynard T, Lebray P, Ingiliz P, Varaud A, Varsat B, Ngo Y, Norha P, Munteanu M, Drane F, Messous D, Imbert Bismut F, Carrau JP, Massard J, Ratziu V, Giordanella JP. Prevalence of liver fibrosis and risk factors in a general population using non-invasive biomarkers (FibroTest). BMC Gastroenterol 2010;10:40.
129. Bonnard P, Sombié R, Lescure FX, Bougouma A, Guiard-Schmid JB, Poynard T, Calès P, Housset C, Callard P, Le Pendeven C, Drabo J, Carrat F, Pialoux G. Comparison of elastography, serum marker scores, and histology for the assessment of liver fibrosis in HBV-infected patients in Burkina Faso. Am J Trop Med Hyg 2010; 82:454-8.
130. Miailhes P, Pradat P, Chevallier M, Lacombe K, Bailly F, Cotte L, Trabaud MA, Boibieux A, Bottero J, Trepo C, Zoulim F. Proficiency of transient elastography compared to liver biopsy for the assessment of fibrosis in HIV/HBV-coinfected patients. J Viral Hepat. 2010 Feb 23.
131. Beckebaum S, Iacob S, Klein CG, Dechêne A, Varghese J, Baba HA, Sotiropoulos GC, Paul A, Gerken G, Cicinnati VR. Assessment of allograft fibrosis by transient elastography and noninvasive biomarker scoring systems in liver transplant patients. Transplantation. 2010;89:983-93.
132. El-Shabrawi MH, Mohsen NA, Sherif MM, El-Karaksy HM, Abou-Yosef H, El-Sayed HM, Riad H, Bahaa N, Isa M, El-Hennawy A. Noninvasive assessment of hepatic fibrosis and necroinflammatory activity in Egyptian children with chronic hepatitis C virus infection using FibroTest and ActiTest. Eur J Gastroenterol Hepatol. 2010 Jan 27.
133. Castera L, Pinzani M. Non-invasive assessment of liver fibrosis: are we ready? Lancet. 2010;375:1419-20.
134. Poynard T, Munteanu M, Ngo Y, Castera L, Halfon P, Ratziu V, Imbert-Bismut F, Thabut D, Bourliere M, Cacoub P, Messous D, de Ledinghen V. ActiTest accuracy for the assessment of histological activity grades in patients with chronic hepatitis C, an overview using Obuchowski measure. Gastroenterol Clin Biol. 2010;34:388-96.
135. Friedrich-Rust M, Rosenberg W, Parkes J, Herrmann E, Zeuzem S, Sarrazin C. Comparison of ELF, FibroTest and FibroScan for the non-invasive assessment of liver fibrosis. BMC Gastroenterol. 2010;10:103.
136. Canbakan M, Senturk H, Canbakan B, Toptas T, Tabak O, Ozaras R, Tabak F, Balcı H, Sut N, Ozbay G. Validation of Biochemical Markers for the Prediction of Liver Fibrosis and Necroinflammatory Activity in Hemodialysis Patients with Chronic Hepatitis C. Nephron Clin Pract. 2010;117:c289-c295.
137. Dominguez S, Ghosn J, Peytavin G, Guiguet M, Tubiana R, Valantin MA, Murphy R, Bricaire F, Benhamou Y, Katlama C. Impact of hepatitis C and liver fibrosis on antiretroviral plasma drug concentrations in HIV-HCV co-infected patients: the HEPADOSE study. J Antimicrob Chemother. 2010;65:2445-9.
138. Moussalli J, Delaquaize H, Boubilley D, Lhomme JP, Merleau Ponty J, Sabot D, Kerever A, Valleur M, Poynard T. Factors to improve the management of hepatitis C in drug users: an observational study in an addiction centre. Gastroenterol Res Pract. 2010;2010.
139. Laharie D, Seneschal J, Schaeverbeke T, Doutre MS, Longy-Boursier M, Pellegrin JL, Chabrun E, Villars S, Zerbib F, de Lédinghen V. Assessment of liver fibrosis with transient elastography and FibroTest in patients treated with methotrexate for chronic inflammatory diseases: a case-control study. J Hepatol. 2010;53:1035-40.
140. Calès P, Boursier J, Bertrais S, Oberti F, Gallois Y, Fouchard-Hubert I, Dib N, Zarski JP, Rousselet MC; multicentric groups (SNIFF 14 & 17, ANRS HC EP 23 Fibrostar). Optimization and robustness of blood tests for liver fibrosis and cirrhosis. Clin Biochem. 2010;43:1315-22.
141. Said Y, Salem M, Mouelhi L, Mekki H, Houissa F, Ben Rejeb M, Moussa A, Trabelsi S, Debbeche R, Bouzaidi S, Najjar T. Correlation between liver biopsy and fibrotest in the evaluation of hepatic fibrosis in patients with chronic hepatitis C. Tunis Med. 2010;88:573-8.
142. Uyar C, Akcam FZ, Ciris M, Kaya O, Kockar C, Isler M. Comparison of FibroTest-ActiTest with histopathology in demonstrating fibrosis and necroinflammatory activity in chronic hepatitis B and C. Indian J Pathol Microbiol. 2010;53:470-5.
143. Poynard T, Ngo Y, Munteanu M, Thabut D, Massard J, Moussalli J, Varaud A, Benhamou Y, Ratziu V. Biomarkers of liver injury for hepatitis clinical trials: a meta-analysis of longitudinal studies. Antivir Ther. 2010;15:617-31.
144. Calès P, Halfon P, Batisse D, Carrat F, Perré P, Penaranda G, Guyader D, d'Alteroche L, Fouchard-Hubert I, Michelet C, Veillon P, Lambert J, Weiss L, Salmon D, Cacoub P. Comparison of liver fibrosis blood tests developed for HCV with new specific tests in HIV/HCV co-infection. J Hepatol. 2010;53:238-44.
145. Diagnosis of fibrosis and cirrhosis. Liver biopsy is not always necessary. Prescrire Int. 2010;19:38-42.
146. Sökücü S, Gökçe S, Güllüoğlu M, Aydoğan A, Celtik C, Durmaz O. The role of the non-invasive serum marker FibroTest-ActiTest in the prediction of histological stage of fibrosis and activity in children with naïve chronic hepatitis B infection. Scand J Infect Dis. 2010;42:699-703.
147. Castéra L, Bernard PH, Le Bail B, Foucher J, Trimoulet P, Merrouche W, Couzigou P, de Lédinghen V. Transient elastography and biomarkers for liver fibrosis assessment and follow-up of inactive hepatitis B carriers. Aliment Pharmacol Ther. 2011;33:455-65.
148. Munteanu M, Hermeziu B, Bismut FI, Poynard T. Performance of the FibroTest cannot be accurately estimated in nine paediatric patients with advanced fibrosis. Scand J Infect Dis. 2011;43:159-60.
149. Zarski JP, Sturm N, Desmorat H, Melin P, Raabe JJ, Bonny C, Sogni P, Pinta A, Rouanet S, Babany G, Cheveau A, Chevallier M. Non-invasive assessment of liver fibrosis progression in hepatitis C patients retreated for 96 weeks with antiviral therapy: a randomized study. Liver Int. 2010;30:1049-58.
150. Ratziu V, de Ledinghen V, Oberti F, Mathurin P, Wartelle-Bladou C, Renou C, Sogni P, Maynard M, Larrey D, Serfaty L, Bonnefont-Rousselot D, Bastard JP, Rivière M, Spénard J; on behalf of the FRESGUN. A randomized controlled trial of high-dose ursodesoxycholic acid for nonalcoholic steatohepatitis. J Hepatol. 2011. In press
151. Poynard T, Munteanu M, Colombo M, Bruix J, Schiff E, Terg R, Flamm S, Moreno-Otero R, Carrilho F, Schmidt W, Berg T, McGarrity T, Heathcote EJ, Gonçales F, Diago M, Craxi A, Silva M, Boparai N, Griffel L, Burroughs M, Brass C, Albrecht J. FibroTest is an independent predictor of virologic response in chronic hepatitis C patients retreated with pegylated interferon alfa-2b and ribavirin in the EPIC3 program. J Hepatol. 2011;54:227-35.
152. Costa JM, Telehin D, Munteanu M, Kobryn T, Ngo Y, Thibault V, Joseph M, RatziuV, Benhamou Y, Koz'ko V, Dubins'ka G, Poveda JD, Poynard T. HCV-GenoFibrotest: A combination of viral, liver and genomic (IL28b, ITPA, UGT1A1) biomarkers for predicting treatment response in patients with chronic hepatitis C. Clin Res Hepatol Gastroenterol. 2011 Feb 25. [Epub ahead of print]
153. Ahmad W, Ijaz B, Gull S, Asad S, Khaliq S, Jahan S, Sarwar MT, Kausar H, Sumrin A, Shahid I, Hassan S. A brief review on molecular, genetic and imaging techniques for HCV fibrosis evaluation. Virol J. 2011;8:53
154. Vergniol J, Foucher J, Terrebonne E, Bernard PH, Le Bail B, Merrouche W, Couzigou P, de Ledinghen V. Non-Invasive Tests for Fibrosis and Liver Stiffness Predict 5-Year Outcomes of Patients with Chronic Hepatitis C. Gastroenterology. 2011 Mar 1. [Epub ahead of print]
155. Terrier B, Carrat F, Geri G, Pol S, Piroth L, Halfon P, Poynard T, Souberbielle JC, Cacoub P. Low 25-OH vitamin D serum levels correlate with severe fibrosis in HIV-HCV co-infected patients with chronic hepatitis. J Hepatol. 2011 Feb 17. [Epub ahead of print]
156. Lassailly G, Caiazzo R, Hollebecque A, Buob D, Leteurtre E, Arnalsteen L, Louvet A, Pigeyre M, Raverdy V, Verkindt H, Six MF, Eberle C, Blanc A, Dharancy S, Romon M, Pattou F, Mathurin P. Validation of non-invasive biomarkers (FibroTest, SteatoTest and NashTest) for prediction of liver injury in patients with morbid obesity
157. Poynard T, Ngo Y, Munteanu M, Thabut D, Ratziu V. Noninvasive Markers of Hepatic Fibrosis in Chronic Hepatitis B. Curr Hepatitis Rep 2011 in press
