# Supplementary material for: Applicability and precautions of use of liver injury biomarker FibroTest. A reappraisal at 7 years of age
Source: BMC Gastroenterol. 2011 Apr 14;11:39. doi: 10.1186/1471-230X-11-39 (PMC3097002; doi:10.1186/1471-230X-11-39)
Supplement: Additional file 2 — Prevalence of samples out of the initial reference range limits (P1, P2, P3). Table S1: Initial reference range for the parameters of FibroTest and ActiTest: Derived from first population definitions (Imbert-Bismut et al Lancet 2001, Myers HBV J Hep, Naveau ALD CGH and Ratziu NAFLD BMC Gastro). Table S2: Normal ranges in apparently healthy volunteers. Blood donors (P1). Distribution using initial references ranges in HCV, HBV, ALD and NAFLD. Table S3: Normal ranges in apparently healthy volunteers. General population P2, FibroTest components performed in the reference center. Table S4: Prevalence of samples out of the initial reference range limits, among global population (P3) [file 1471-230X-11-39-S2.DOCX]

**Additional File 2: Prevalence of samples out of the initial reference range limits (P1, P2, P3)**

**Additional file 2, Table S1: Initial reference range for the parameters of FibroTest and ActiTest : Derived from first population definitions (Imbert-Bismut et al Lancet 2001, Myers HBV J Hep, Naveau ALD CGH and Ratziu NAFLD BMC Gastro)**

| **Parameters** | **Lower limit**  **n (%)** | **Upper limit**  **n (%)** |
| --- | --- | --- |
| **Alpha-2 macroglobulin g/L** | <=0.80 (1%) | >=5.90 (1%) |
| **Apolipoprotein A1 g/L** | <=0.56 (1%) | >=2.50 (1%) |
| **Haptoglobin g/L** | <=0.08 (1%) | >=3.20 (1%) |
| **GGT IU/L** | <1 (<1%) | >=1140 (1%) |
| **Total Bilirubin, µmol/L** | <1 (<1%) | >=50.00 (1%) |
| **ALT IU/L** | <1 (<1%) | >622 (1%) |

**Additional file 2, Table S2: Normal ranges in apparently healthy volunteers. Blood donors (P1).**

**Distribution using initial references ranges in HCV, HBV, ALD and NAFLD**

| **Parameters**  **Blood donors N=954** | **Lower limit**  **n (%)** | **Upper limit**  **n (%)** | **Total out of the limits, n (%)** | **In the limits, n (%)** |
| --- | --- | --- | --- | --- |
| **Alpha-2 macroglobulin g/L** | 4 (0.40%) | 0 | 4 (0.40%) | 950 (99.60%) |
| **Apolipoprotein A1 g/L** | 4 (0.40%) | 0 | 4 (0.40%) | 950 (99.60%) |
| **Haptoglobin g/L** | 6 (0.60%) | 0 | 6 (0.60%) | 948 (99.40%) |
| **GGT IU/L** | 0 | 0 | 0 | 954 (100%) |
| **Total Bilirubin, µmol/L** | 0 | 0 | 0 | 954 (100%) |

**Additional file 2, Table S3: Normal ranges in apparently healthy volunteers. General population P2, FibroTest components performed in the reference center**

| **Parameters**  **General population N=7,457^1^** | **Lower limit**  **n (%)** | **Upper limit**  **n (%)** | **Total out of the limits, n (%)** | **In the limits, n (%)** |
| --- | --- | --- | --- | --- |
| **Alpha-2 macroglobulin g/L** | 67 (0.89%) | 0 | 67 (0.89%) | 7487 (99.11%) |
| **Apolipoprotein A1 g/L** | 1 (0.01%) | 55 (0.73%) | 56 (0.74%) | 7498 (99.26%) |
| **Haptoglobin g/L** | 48 (0.64%) | 25 (0.33%) | 73 (0.97%) | 7481 (99.03%) |
| **GGT IU/L** | 0 | 1 (0.01%) | 1 (0.01%) | 7553 (99,99%) |
| **Total Bilirubin, µmol/L** | 0 | 0 | 0 | 7554 (100%) |

**Additional file 2, Table S4: Prevalence of samples out of the initial reference range limits, among global population (P3)**

| **Parameters N=345,695** | **Lower limit n (%)** | **Upper limit n (%)** |
| --- | --- | --- |
| **Haptoglobin g/L** | 6046 (1.75%) | 2202 (0.64%) |
| **Apolipoprotein A1 g/L** | 2603 (0.75%) | 2136 (0.62%) |
| **Alpha-2 macroglobulin g/L** | 703 (0.20%) | 712 (0.21%) |
| **GGT IU/L** | 0 (0%) | 136 (0.04%) |
| **Total Bilirubin, µmol/L** | 0 (0%) | 29 (0.01%) |
