# Supplementary material for: Applicability and precautions of use of liver injury biomarker FibroTest. A reappraisal at 7 years of age
Source: BMC Gastroenterol. 2011 Apr 14;11:39. doi: 10.1186/1471-230X-11-39 (PMC3097002; doi:10.1186/1471-230X-11-39)
Supplement: Additional file 3 — Factors associated with high risk profile in patients' global population (P3). Tables S5: Alpha2 macroglobulin. Table S6: Apolipoprotein A1. Table S7: High haptoglobin and High GGT [file 1471-230X-11-39-S3.DOCX]

**Additional File 3: Factors associated with high risk profile in patients' global population (P3)**

**Additional file 3, Table S5: Alpha2 macroglobulin**

|  | **Alpha2 macroglobulin** | | | | | |  | |
| --- | --- | --- | --- | --- | --- | --- | --- | --- |
|  | **False Positive** % **(Odds Ratio) n=427** | | **False Negative n=419** | | |  |  | |
| **Range** | **5.90-9.68 g/L** | | | | **0.10-0.80 g/L** |  |  | |
| **Factor (n)** | **Univariate** | **Multivariate** | | **Univariate** | | **Multivariate** |  |  |
| Age > 50 years | 1.43 (1.18-1.74) P=0.0002 | 1.56 (1.29-1.90) P=10^-4^ | | 1.26 (1.03-1.53) P=0.02 | | 1.45 (1.19-1.76) P=0.0002 | |  |
| Male gender | 1.20 (0.98-1.47) P=0.07 | 1.34 (1.10-1.63) P=0.004 | | 2.48 (1.97-3.15) P<10^-5^ | | 2.6 (2.07-3.28) P<10^-5^ | |  |
| Continent |  |  | |  | |  | |  |
| Eastern Europe | NS | 6.63 (2.08-21.16) P=0.001 | | NS | | 22.44 (5.55-90.85) P<10^-4^ | |  |
| >10,000 tests | NS | NS | | NS | | NS | |  |
| Last 3 years of test | NS | NS | | NS | | NS | |  |
| Reference center | NS | NS | | 1.92 (1.42-2.56) P=10^-4^ | | 1.83 (1.36- 2.47) P=0.00008 | |  |
| HIV center | NS | NS | | NS | | NS | |  |

**Additional file 3, Table S6: Apolipoprotein A1**

|  | **Apolipoprotein A1** | | |  | | | |
| --- | --- | --- | --- | --- | --- | --- | --- |
|  | **False Positive** % **(Odds Ratio) n=118** | | | **False Negative n=732** | | |  |
| **Range** | **0.10-0.41 g/L** |  | **2.51-6.97 g/L** | | |  | |
| **Factor (n)** | **Univariate** | **Multivariate** | **Univariate** | | **Multivariate** | | |
| Age > 50 years | 1.98 (1.34-2.95) P=0.0003 | 2.01 (1.38-2.94) | 2.06 (1.77-2.39) P<10^-5^ | | 1.86 (1.60-1.86) P<10^-5^ | | |
| Male gender | NS | NS | 0.30 (0.25-0.35) P<10^-5^ | | 0.34 (0.29-0.40) P<10^-5^ | | |
| Continent |  |  |  | |  | | |
| Eastern Europe | NS | NS | NS | | 22.44 (5.55-90.85) P<10^-4^ | | |
| >10,000 tests | 2.73 (1.84-4.08) P<10^-5^ | 2.31 (1.56-3.42) P=0.0003 | 0.46 (0.39-0.55) P<10^-5^ | | 0.48 ((0.39-0.50) P<10^-5^ | | |
| Last 3 years of test | NS | NS | 0.89 (0.81-0.98) P=0.02 | | 1.26 (1.07-1.49) P=0.005 | | |
|  |  |  |  | |  | | |
| Reference center | 3.50 (2.18-5.50) P<10^-5^ | 2.61 (1.66-4.13) P=0.00003 | 1.09 (0.82-1.44) P=0.58 | | 1.84 (1.39- 2.45) P<10^-5^ | | |
| HIV center | NS | NS | NS | | NS | | |

**Additional file 3, Table S7: low haptoglobin and high GGT**

|  | **Haptoglobin** | | **GGT** | |
| --- | --- | --- | --- | --- |
|  | **False Positive** % **(Odds Ratio) n=1,590** |  | **False Positive n=78** |  |
| **Range** | **0.01-0.08** | **R^2^=0.76** |  |  |
| **Factor (n)** | **Univariate** | **Multivariate** | **Univariate** | **Multivariate** |
| Continent |  |  |  | NS |
| Western Europe | 0.40 NS | 0.38 (0.22-0.64) P=0.0004 | 0.03 |  |
| North Africa | 0.28 NS | 0.24 (0.12-0.51) P=0.0002 | 0.00 |  |
| North America | 0.03 NS | 0.05 (0.01-0.20) P=0.00005 | 0.01 |  |
| >10,000 tests | 0.4 NS | 0.44 ((0.39-0.50) P<10^-5^ | NS | NS |
| Last 3 years of test | 0.89 (0.81-0.98) P=0.02 | 0.76 (0.68-0.84) P<10^-5^ | NS |  |
| Reference center | 3.33 (2.94-3.77) P<10^-4^ | 5.35 (4.67- 6.13) P<10^-5^ | NS | NS |
| HIV center | 3.55 (2.55-4.87) P<10^-4^ | 3.97 (2.88-5.48) P<10^-5^ | NS | NS |
| Age > 50 years | 0.63 (0.57-0.70) P<10-4 | 0.69 (0.63-0.77) P<10-5 | NS | NS |
| Male gender | 1.30 (1.18-1.45) P<10-4 | 1.12 (1.01-1.25) P=0.03 | NS | NS |
