# Supplementary material for: Applicability and precautions of use of liver injury biomarker FibroTest. A reappraisal at 7 years of age
Source: BMC Gastroenterol. 2011 Apr 14;11:39. doi: 10.1186/1471-230X-11-39 (PMC3097002; doi:10.1186/1471-230X-11-39)
Supplement: Additional file 4 — Patients details among patients of the reference tertiary care center (P4). [file 1471-230X-11-39-S4.DOCX]

**Additional File 4: Patients details among patients of the reference tertiary care center (P4)**

Additional File 4, Table S8: Low haptoglobin (<=0.08 g/L) among 214 inpatients of reference center (P4)

| Id_Final | Sexe | Hapto | Age | Main diagnosis | Ascites | Fibrotest | Expc | HIV |
| --- | --- | --- | --- | --- | --- | --- | --- | --- |
| 114 | 1 | 0,08 | 43 | HIV, Homozygote hemochromatosis C282 | 0 | 0.72 F3F4 | TP | HIV |
| 108 | 0 | 0,07 | 46 | Chronic hepatitis C, HIV | 0 | 0.81 F4 | TP | HIV |
| 108 | 0 | 0,07 | 48 | Chronic hepatitis C, HIV | 0 | 0.75 F4 | TP | HIV |
| 108 | 0 | 0,07 | 48 | Chronic hepatitis C, HIV | 0 | 0.77 F4 | TP | HIV |
| 102 | 1 | 0,07 | 47 | Chronic hepatitis C, HIV | 0 | 0.75 F4 | TP | HIV |
| 102 | 1 | 0,08 | 47 | Chronic hepatitis C, HIV | 0 | 0.63 F3 | TP | HIV |
| 102 | 1 | 0,07 | 49 | Chronic hepatitis C, HIV | 0 | 0.58 F2 | TP | HIV |
| 95 | 0 | 0,08 | 62 | Chronic hepatitis C, HIV | - | 0.64 F3 | TP | HIV |
| 86 | 0 | 0,08 | 38 | Chronic hepatitis C HIV | 0 | 0.62 F3 | TP | HIV |
| 86 | 0 | 0,06 | 40 | Chronic hepatitis C HIV | 0 | 0.85 F4 | TP | HIV |
| 106 | 1 | 0,01 | 38 | Chronic hepatitis B, HIV | 0 | 0.81 F4 | TP | HIV |
| 27 | 1 | 0,07 | 50 | Chronic hepatitis B HIV NAFLD | 0 | 0.82 F4 | TP | HIV |
| 27 | 1 | 0,07 | 50 | Chronic hepatitis B HIV NAFLD | 0 | 0.79 F4 | TP | HIV |
| 30 | 1 | 0,07 | 39 | Chronic hepatitis B HIV | 0 | 0.65 F3 | TP | HIV |
| 30 | 1 | 0,07 | 40 | Chronic hepatitis B HIV | 0 | 0.60 F3 | TP | HIV |
| 53 | 1 | 0,07 | 45 | Chronic hepatitis B HIV | 0 | 0.70 F3 | TP | HIV |
| 53 | 1 | 0,07 | 46 | Chronic hepatitis B HIV | 0 | 0.69 F3 | TP | HIV |
| 53 | 1 | 0,07 | 47 | Chronic hepatitis B HIV | 0 | 0.63 F3 | TP | HIV |
| 83 | 1 | 0,08 | 43 | SVR hepatitis C | 0 | 0.64 F3 | TP |  |
| 25 | 1 | 0,07 | 42 | OV Rupture | 0 | 0.63 F3 | TP |  |
| 21 | 1 | 0,08 | 54 | OV Rupture | 0 | 0.80 F4 | TP |  |
| 18 | 0 | 0,08 | 60 | OV Rupture | 0 | 0.79 F4 | TP |  |
| 7 | 1 | 0,07 | 19 | Other: cholestasis/ bilharziosis in renal transplanted | 0 | 0.55 F2 | TP |  |
| 46 | 1 | 0,07 | 50 | Other (cirrhosis "toxic" origin) | 0 | 0.79 F4 | TP |  |
| 92 | 1 | 0,06 | 42 | NAFLD, double heterozygote for HFE gene | 0 | 0.81 F4 | TP |  |
| 92 | 1 | 0,07 | 44 | NAFLD, double heterozygote for HFE gene | 0 | 0.82 F4 | TP |  |
| 36 | 1 | 0,07 | 62 | NAFLD ALD Other(HNR, Heterozygote Hemochromatosis ) | 0 | 0.76 F4 | TP |  |
| 36 | 1 | 0,07 | 62 | NAFLD ALD Other(HNR, Heterozygote Hemochromatosis ) | 0 | 0.76 F4 | TP |  |
| 36 | 1 | 0,07 | 62 | NAFLD ALD Other(HNR, Heterozygote Hemochromatosis ) | 0 | 0.76 F4 | TP |  |
| 89 | 1 | 0,01 | 50 | Cirrhosis HBV | 0 | 0.94 F4 | TP |  |
| 118 | 0 | 0,01 | 55 | Cirhosis HCV, ALD | 0 | 0.82 F4 | TP |  |
| 97 | 0 | 0,01 | 61 | Cihhrosis HBV | 0 | 0.72 F3 | TP |  |
| 70 | 1 | 0,07 | 40 | Chronic hepatitis C NASH | 0 | 0.80 F4 | TP |  |
| 70 | 1 | 0,06 | 42 | Chronic hepatitis C NASH | 0 | 0.85 F4 | TP |  |
| 70 | 1 | 0,08 | 42 | Chronic hepatitis C NASH | 0 | 0.74 F3F4 | TP |  |
| 70 | 1 | 0,07 | 42 | Chronic hepatitis C NASH | 0 | 0.82 F4 | TP |  |
| 70 | 1 | 0,07 | 43 | Chronic hepatitis C NASH | 0 | 0.80 F4 | TP |  |
| 47 | 0 | 0,08 | 46 | Chronic hepatitis C AIH | 0 | 0.72 F3F4 | TP |  |
| 47 | 0 | 0,08 | 46 | Chronic hepatitis C AIH | 0 | 0.74 F3F4 | TP |  |
| 47 | 0 | 0,08 | 47 | Chronic hepatitis C AIH | 0 | 0.67 F3 | TP |  |
| 47 | 0 | 0,08 | 47 | Chronic hepatitis C AIH | 0 | 0.68 F3 | TP |  |
| 123 | 0 | 0,07 | 46 | Chronic hepatitis C | 0 | 0.81 F4 | TP |  |
| 3 | 0 | 0,07 | 49 | Chronic hepatitis C | 0 | 0.74 F3F4 | TP |  |
| 3 | 0 | 0,07 | 52 | Chronic hepatitis C | 0 | 0.81 F4 | TP |  |
| 3 | 0 | 0,07 | 52 | Chronic hepatitis C | 0 | 0.81 F4 | TP |  |
| 26 | 0 | 0,01 | 19 | Chronic hepatitis C | 0 | 0.84 F4 | TP |  |
| 26 | 0 | 0,01 | 19 | Chronic hepatitis C | 0 | 0.17 F4 | TP |  |
| 9 | 1 | 0,07 | 39 | Chronic hepatitis B Delta | 0 | 0.84 F4 | TP |  |
| 5 | 1 | 0,06 | 39 | Chronic hepatitis B | 0 | 0.81 F4 | TP |  |
| 5 | 1 | 0,08 | 39 | Chronic hepatitis B | 0 | 0.63 F3 | TP |  |
| 68 | 1 | 0,08 | 50 | Chronic hepatitis B | 0 | 0.79 F4 | TP |  |
| 8 | 1 | 0,07 | 60 | Chronic hepatitis B | 0 | 0.68 F3 | TP |  |
| 80 | 0 | 0,08 | 47 | Chronic hepatitis B | 0 | 0.56 F2 | TP |  |
| 112 | 1 | 0,08 | 36 | Chronic hepatitis B | 0 | 0.78 F4 | TP |  |
| 63 | 1 | 0,07 | 35 | Chronic hepatitis B | 0 | 0.52 F2 | TP |  |
| 1 | 0 | 0,08 | 64 | ALD | 0 | 0.62 F3 | TP |  |
| 82 | 1 | 0,08 | 60 | ALD | unknown | 0.71 F3 | TP |  |
| 82 | 1 | 0,08 | 60 | ALD | unknown | 0.71 F3 | TP |  |
| 117 | 1 | 0,07 | 33 | HIV | 0 | 0.79 F4 | Ind | HIV |
| 94 | 0 | 0,07 | 51 | Chronic hepatitis C, HIV | 0 | 0.79 F4 | Ind | HIV |
| 105 | 1 | 0,08 | 39 | Chronic hepatitis B, HIV | 0 | 0.54 F2 | Ind | HIV |
| 105 | 1 | 0,07 | 43 | Chronic hepatitis B, HIV | 0 | 0.58 F3 | Ind | HIV |
| 105 | 1 | 0,07 | 44 | Chronic hepatitis B, HIV | 0 | 0.59 F3 | Ind | HIV |
| 40 | 1 | 0,08 | 36 | Chronic hepatitis B HIV | 0 | 0.59 F3 | Ind | HIV |
| 40 | 1 | 0,08 | 36 | Chronic hepatitis B HIV | 0 | 0.59 F3 | Ind | HIV |
| 24 | 1 | 0,08 | 47 | Chronic hepatitis B HIV | 0 | 0.69 F3 | Ind | HIV |
| 24 | 1 | 0,08 | 47 | Chronic hepatitis B HIV | 0 | 0.57 F2 | Ind | HIV |
| 81 | 1 | 0,08 | 28 | Chronic hepatitis C HIV | 0 | 0.49 F2 | Ind | HIV |
| 99 | 0 | 0,07 | 68 | SVR hepatitis C | 0 | 0.47 F1F2 | Ind |  |
| 87 | 1 | 0,08 | 53 | Liver metastasis | 0 | 0.76 F4 | Ind |  |
| 87 | 1 | 0,08 | 53 | Liver metastasis | 0 | 0.76 F4 | Ind |  |
| 87 | 1 | 0,08 | 53 | Liver metastasis | 0 | 0.76 F4 | Ind |  |
| 14 | 1 | 0,07 | 33 | Chronic hepatitis C | 0 | 0.53 F2 | Ind |  |
| 19 | 0 | 0,07 | 38 | Chronic hepatitis C | 0 | 0.79 F4 | Ind |  |
| 15 | 0 | 0,01 | 57 | Chronic hepatitis C | 0 | 0.87 F4 | Ind |  |
| 2 | 1 | 0,07 | 38 | Chronic hepatitis C | 0 | 0.73 F3F4 | Ind |  |
| 74 | 0 | 0,08 | 64 | Chronic hepatitis C | 0 | 0.70 F3 | Ind |  |
| 91 | 0 | 0,08 | 53 | Chronic hepatitis C | 0 | 0.68 F3 | Ind |  |
| 119 | 0 | 0,07 | 30 | Chronic hepatitis C | 0 | 0.51 F2 | Ind |  |
| 51 | 0 | 0,07 | 50 | Chronic hepatitis C | 0 | 0.68 F3 | Ind |  |
| 44 | 0 | 0,08 | 53 | Chronic hepatitis C | 0 | 0.54 F2 | Ind |  |
| 61 | 1 | 0,07 | 22 | Chronic hepatitis B Delta | 0 | 0.67 F3 | Ind |  |
| 31 | 1 | 0,07 | 46 | Chronic hepatitis B and HCC | 0 | 0.82 F4 | Ind |  |
| 41 | 1 | 0,08 | 29 | Chronic hepatitis B | 0 | 0.55 F2 | Ind |  |
| 113 | 1 | 0,08 | 24 | Chronic hepatitis B | 0 | 0.51 F2 | Ind |  |
| 79 | 1 | 0,07 | 28 | Chronic hepatitis B | 0 | 0.55 F2 | Ind |  |
| 71 | 1 | 0,07 | 33 | Chronic hepatitis B | 0 | 0.50 F2 | Ind |  |
| 29 | 1 | 0,01 | 32 | Chronic hepatitis B | 0 | 0.80 F4 | Ind |  |
| 17 | 1 | 0,07 | 35 | Chronic hepatitis B | 0 | 0.70 F3 | Ind |  |
| 111 | 1 | 0,08 | 31 | Chronic hepatitis B | 0 | 0.70 F3 | Ind |  |
| 104 | 1 | 0,08 | 34 | Chronic hepatitis B | 0 | 0.65 F3 | Ind |  |
| 116 | 0 | 0,08 | 40 | Chronic hepatitis B | 0 | 0.53 F2 | Ind |  |
| 121 | 1 | 0,07 | 50 | Chronic hepatitis B | 0 | 0.72 F3 | Ind |  |
| 115 | 1 | 0,01 | 38 | Chronic hepatitis B | 0 | 0.93 F4 | Ind |  |
| 115 | 1 | 0,07 | 39 | Chronic hepatitis B | 0 | 0.77 F4 | Ind |  |
| 35 | 1 | 0,07 | 37 | Chronic hepatitis B | 0 | 0.69 F3 | Ind |  |
| 50 | 1 | 0,07 | 29 | Chronic hepatitis B | 0 | 0.53 F2 | Ind |  |
| 50 | 1 | 0,07 | 32 | Chronic hepatitis B | 0 | 0.49 F2 | Ind |  |
| 45 | 1 | 0,06 | 36 | ALD | 0 | 0.73 F3F4 | Ind |  |
| 85 | 1 | 0,07 | 59 | abnormal liver enzymes | 0 | 0.62 F3 | Ind |  |
| 96 | 1 | 0,08 | 51 | HIV, Episode of acute hepatitis C in resolution | 0 | 0.69 F3 | FP | HIV |
| 98 | 0 | 0,08 | 45 | Chronic hepatitis C, HIV, ALD | 0 | 0.65 F3 | FP | HIV |
| 98 | 0 | 0,08 | 45 | Chronic hepatitis C, HIV, ALD | 0 | 0.68 F3 | FP | HIV |
| 78 | 1 | 0,08 | 42 | Chronic hepatitis C HIV | 0 | 0.61 F3 | FP | HIV |
| 78 | 1 | 0,01 | 42 | Chronic hepatitis C HIV | 0 | 0.85 F4 | FP | HIV |
| 78 | 1 | 0,01 | 42 | Chronic hepatitis C HIV | 0 | 0.84 F4 | FP | HIV |
| 69 | 1 | 0,07 | 42 | Chronic hepatitis C HIV | 0 | 0.55 F2 | FP | HIV |
| 65 | 1 | 0,08 | 40 | Chronic hepatitis C HIV | 0 | 0.56 F2 | FP | HIV |
| 65 | 1 | 0,07 | 42 | Chronic hepatitis C HIV | 0 | 0.47 F1F2 | FP | HIV |
| 32 | 1 | 0,08 | 34 | Chronic hepatitis B Delta, HIV | 0 | 0.81 F4 | FP | HIV |
| 88 | 1 | 0,01 | 66 | ALD HIV | 0 | 0.78 F4 | FP | HIV |
| 16 | 1 | 0,07 | 53 | Portal Thrombosis | 0 | 0.68 F3 | FP |  |
| 10 | 1 | 0,07 | 38 | NAFLD | 0 | 0.59 F3 | FP |  |
| 10 | 1 | 0,07 | 39 | NAFLD | 0 | 0.66 F3 | FP |  |
| 10 | 1 | 0,07 | 39 | NAFLD | 0 | 0.65 F3 | FP |  |
| 10 | 1 | 0,07 | 40 | NAFLD | 0 | 0.69 F3 | FP |  |
| 10 | 1 | 0,07 | 40 | NAFLD | 0 | 0.69 F3 | FP |  |
| 10 | 1 | 0,08 | 40 | NAFLD | 0 | 0.70 F3 | FP |  |
| 10 | 1 | 0,08 | 41 | NAFLD | 0 | 0.66 F3 | FP |  |
| 10 | 1 | 0,08 | 41 | NAFLD | 0 | 0.63 F3 | FP |  |
| 10 | 1 | 0,01 | 38 | NAFLD | 0 | 0.63 F3 | FP |  |
| 56 | 1 | 0,07 | 52 | NAFLD | 0 | 0.52 F2 | FP |  |
| 38 | 1 | 0,07 | 60 | NAFLD | 0 | 0.83 F4 | FP |  |
| 107 | 1 | 0,07 | 54 | Chronic hepatitis C, twice renal transplanted | 0 | 0.77 F4 | FP |  |
| 109 | 0 | 0,08 | 58 | Chronic hepatitis C and B | 0 | 0.68 F3 | FP |  |
| 66 | 1 | 0,08 | 56 | Chronic hepatitis C Castelman disease | 0 | 0.60 F3 | FP |  |
| 76 | 1 | 0,08 | 72 | Chronic hepatitis C | 0 | 0.62 F3 | FP |  |
| 76 | 1 | 0,07 | 74 | Chronic hepatitis C | 0 | 0.72 F3F4 | FP |  |
| 54 | 1 | 0,07 | 49 | Chronic hepatitis C | 0 | 0.74 F4 | FP |  |
| 58 | 1 | 0,08 | 53 | Chronic hepatitis C | 0 | 0.80 F4 | FP |  |
| 43 | 0 | 0,08 | 34 | Chronic hepatitis C | 0 | 0.66 F3 | FP |  |
| 43 | 0 | 0,01 | 35 | Chronic hepatitis C | 0 | 0.85 F4 | FP |  |
| 33 | 1 | 0,08 | 45 | Chronic hepatitis C | 0 | 0.57 F2 | FP |  |
| 33 | 1 | 0,07 | 48 | Chronic hepatitis C | 0 | 0.67 F3 | FP |  |
| 28 | 1 | 0,08 | 43 | Chronic hepatitis C | 0 | 0.74 F4 | FP |  |
| 28 | 1 | 0,08 | 44 | Chronic hepatitis C | 0 | 0.74 F4 | FP |  |
| 28 | 1 | 0,07 | 44 | Chronic hepatitis C | 0 | 0.68 F3 | FP |  |
| 28 | 1 | 0,07 | 47 | Chronic hepatitis C | 0 | 0.75 F4 | FP |  |
| 75 | 0 | 0,08 | 72 | Chronic hepatitis C | 0 | 0.80 F4 | FP |  |
| 23 | 1 | 0,07 | 74 | Chronic hepatitis C | 0 | 0.77 F4 | FP |  |
| 52 | 0 | 0,07 | 43 | Chronic hepatitis C | 0 | 0.73 F3F4 | FP |  |
| 20 | 0 | 0,08 | 58 | Chronic hepatitis C | 0 | 0.79 F4 | FP |  |
| 20 | 0 | 0,08 | 58 | Chronic hepatitis C | 0 | 0.79 F4 | FP |  |
| 11 | 1 | 0,01 | 30 | Chronic hepatitis C | 0 | 0.78 F4 | FP |  |
| 22 | 0 | 0,08 | 49 | Chronic hepatitis C | 0 | 0.59 F3 | FP |  |
| 22 | 0 | 0,07 | 50 | Chronic hepatitis C | 0 | 0.72 F3 | FP |  |
| 22 | 0 | 0,07 | 50 | Chronic hepatitis C | 0 | 0.76 F4 | FP |  |
| 22 | 0 | 0,07 | 51 | Chronic hepatitis C | 0 | 0.66 F3 | FP |  |
| 4 | 0 | 0,08 | 52 | Chronic hepatitis C | 0 | 0.70 F3 | FP |  |
| 120 | 1 | 0,07 | 51 | Chronic hepatitis C | 0 | 0.82 F4 | FP |  |
| 100 | 0 | 0,07 | 59 | Chronic hepatitis C | - | 0.83 F4 | FP |  |
| 37 | 0 | 0,07 | 70 | Chronic hepatitis C | 0 | 0.61 F3 | FP |  |
| 84 | 0 | 0,07 | 36 | Chronic hepatitis C | 0 | 0.77 F4 | FP |  |
| 84 | 0 | 0,07 | 36 | Chronic hepatitis C | 0 | 0.17 F4 | FP |  |
| 60 | 0 | 0,08 | 73 | Chronic hepatitis C | 0 | 0.75 F4 | FP |  |
| 101 | 0 | 0,08 | 46 | Chronic hepatitis C | 0 | 0.70 F3 | FP |  |
| 110 | 1 | 0,08 | 44 | Chronic hepatitis C | 0 | 0.58 F2 | FP |  |
| 110 | 1 | 0,07 | 45 | Chronic hepatitis C | 0 | 0.62 F3 | FP |  |
| 110 | 1 | 0,08 | 46 | Chronic hepatitis C | 0 | 0.66 F3 | FP |  |
| 110 | 1 | 0,06 | 46 | Chronic hepatitis C | 0 | 0.65 F3 | FP |  |
| 110 | 1 | 0,08 | 47 | Chronic hepatitis C | 0 | 0.69 F3 | FP |  |
| 110 | 1 | 0,07 | 48 | Chronic hepatitis C | 0 | 0.64 F3 | FP |  |
| 57 | 1 | 0,07 | 52 | Chronic hepatitis C | 0 | 0.52 F2 | FP |  |
| 72 | 1 | 0,07 | 41 | Chronic hepatitis B and Delta | 0 | 0.69 F3 | FP |  |
| 48 | 1 | 0,06 | 34 | Chronic hepatitis B | 0 | 0.63 F3 | FP |  |
| 48 | 1 | 0,07 | 35 | Chronic hepatitis B | 0 | 0.63 F3 | FP |  |
| 67 | 1 | 0,07 | 53 | Chronic hepatitis B | 0 | 0.77 F4 | FP |  |
| 13 | 1 | 0,07 | 37 | Chronic hepatitis B | 0 | 0.68 F3 | FP |  |
| 13 | 1 | 0,08 | 33 | Chronic hepatitis B | 0 | 0.64 F3 | FP |  |
| 13 | 1 | 0,08 | 34 | Chronic hepatitis B | 0 | 0.60 F3 | FP |  |
| 13 | 1 | 0,01 | 35 | Chronic hepatitis B | 0 | 0.70 F3 | FP |  |
| 13 | 1 | 0,07 | 36 | Chronic hepatitis B | 0 | 0.72 F3 | FP |  |
| 13 | 1 | 0,07 | 37 | Chronic hepatitis B | 0 | 0.62 F3 | FP |  |
| 13 | 1 | 0,07 | 38 | Chronic hepatitis B | 0 | 0.69 F3 | FP |  |
| 34 | 1 | 0,08 | 49 | Chronic hepatitis B | 0 | 0.57 F2 | FP |  |
| 34 | 1 | 0,01 | 50 | Chronic hepatitis B | 0 | 0.58 F3 | FP |  |
| 34 | 1 | 0,01 | 50 | Chronic hepatitis B | 0 | 0.58 F3 | FP |  |
| 34 | 1 | 0,07 | 51 | Chronic hepatitis B | 0 | 0.60 F3 | FP |  |
| 34 | 1 | 0,06 | 52 | Chronic hepatitis B | 0 | 0.66 F3 | FP |  |
| 34 | 1 | 0,08 | 52 | Chronic hepatitis B | 0 | 0.53 F2 | FP |  |
| 34 | 1 | 0,07 | 53 | Chronic hepatitis B | 0 | 0.59 F3 | FP |  |
| 34 | 1 | 0,07 | 54 | Chronic hepatitis B | 0 | 0.65 F3 | FP |  |
| 64 | 1 | 0,08 | 59 | Chronic hepatitis B | 0 | 0.66 F3 | FP |  |
| 64 | 1 | 0,08 | 61 | Chronic hepatitis B | 0 | 0.56 F2 | FP |  |
| 64 | 1 | 0,07 | 61 | Chronic hepatitis B | 0 | 0.57 F2 | FP |  |
| 64 | 1 | 0,08 | 63 | Chronic hepatitis B | 0 | 0.74 F3F4 | FP |  |
| 49 | 1 | 0,08 | 35 | Chronic hepatitis B | 0 | 0.81 F4 | FP |  |
| 49 | 1 | 0,08 | 35 | Chronic hepatitis B | 0 | 0.75 F4 | FP |  |
| 49 | 1 | 0,08 | 37 | Chronic hepatitis B | 0 | 0.75 F4 | FP |  |
| 42 | 1 | 0,07 | 28 | Chronic hepatitis B | 0 | 0.49 F2 | FP |  |
| 122 | 0 | 0,07 | 51 | Chronic hepatitis B | 0 | 0.69 F3 | FP |  |
| 55 | 1 | 0,08 | 45 | Chronic hepatitis B | 0 | 0.62 F3 | FP |  |
| 55 | 1 | 0,08 | 45 | Chronic hepatitis B | 0 | 0.57 F2 | FP |  |
| 55 | 1 | 0,08 | 45 | Chronic hepatitis B | 0 | 0.57 F2 | FP |  |
| 55 | 1 | 0,08 | 46 | Chronic hepatitis B | 0 | 0.63 F3 | FP |  |
| 55 | 1 | 0,07 | 46 | Chronic hepatitis B | 0 | 0.53 F2 | FP |  |
| 55 | 1 | 0,06 | 47 | Chronic hepatitis B | 0 | 0.70 F3 | FP |  |
| 39 | 1 | 0,08 | 38 | Chronic hepatitis B | 0 | 0.60 F3 | FP |  |
| 39 | 1 | 0,08 | 39 | Chronic hepatitis B | 0 | 0.69 F3 | FP |  |
| 39 | 1 | 0,08 | 40 | Chronic hepatitis B | 0 | 0.71 F3 | FP |  |
| 39 | 1 | 0,08 | 41 | Chronic hepatitis B | 0 | 0.74 F3F4 | FP |  |
| 39 | 1 | 0,07 | 42 | Chronic hepatitis B | 0 | 0.75 F4 | FP |  |
| 6 | 1 | 0,08 | 27 | Chronic hepatitis B | 0 | 0.76 F4 | FP |  |
| 103 | 1 | 0,01 | 26 | Chronic hepatitis B | 0 | 0.81 F4 | FP |  |
| 93 | 0 | 0,01 | 67 | Chronic hepatitis B | 0 | 0.80 F4 | FP |  |
| 73 | 1 | 0,08 | 41 | Chronic hepatitis B | 0 | 0.70 F3 | FP |  |
| 73 | 1 | 0,08 | 41 | Chronic hepatitis B | 0 | 0.70 F3 | FP |  |
| 59 | 1 | 0,07 | 72 | ALD NAFLD | 0 | 0.82 F4 | FP |  |
| 62 | 0 | 0,08 | 72 | ALD | 0 | 0.56 F2 | FP |  |
| 62 | 0 | 0,01 | 73 | ALD | 0 | 0.68 F3 | FP |  |
| 77 | 1 | 0,07 | 53 | ALD | 0 | 0.70 F3 | FP |  |
| 77 | 1 | 0,07 | 54 | ALD | 0 | 0.72 F3F4 | FP |  |
| 90 | 0 | 0,07 | 37 | abnormal liver enzymes post renal transplantation | 0 | 0.82 F4 | FP |  |
| 12 | 0 | 0,08 | 52 | abnormal liver enzymes | 0 | 0.51 F2 | FP |  |
