# Supplementary material for: Applicability and precautions of use of liver injury biomarker FibroTest. A reappraisal at 7 years of age
Source: BMC Gastroenterol. 2011 Apr 14;11:39. doi: 10.1186/1471-230X-11-39 (PMC3097002; doi:10.1186/1471-230X-11-39)
Supplement: Additional file 5 — Patients details among patients of the reference tertiary care center (P4) other than low haptoglobin. Table S8: Low haptoglobin (< = 0.08 g/L) among 214 inpatients of reference center (P4). Table S9: High GGT > = 1140. Table S10: Low Apoa1 < = 0.56. Table S11: High A2M > = 5.90. Table S12: Low A2M (< = 0.80 g/L). Table S13: High Apoa1 > = 2.50. [file 1471-230X-11-39-S5.DOCX]

**Additional File 5: Patients details among patients of the reference tertiary care center (P4) other than low Haptoglobin**

**Additional File 5, Table S9: High GGT >= 1140**

|  | 77092 | 96556 | 31340 | **298321 (x2)** | **299959** |
| --- | --- | --- | --- | --- | --- |
| Factor |  |  |  |  |  |
| Age (year) | 62 | 32 | 50 | 56 | 72 |
| Gender | M | M | M | M | F |
| Continent | W Europe | Asia | W Europe | W Europe | W Europe |
| GGT (IU/L) | 2885 | 1561 | 3586 | 4722 | 2543 |
| Proteins (g/L) | 48 | 78 | 76 | NA | NA |
| Main diagnosis | Duodenal Ulcer perforation | HIV | ALD, Tuberculosis Chronic pancreatitis | Multiple myeloma bone marrow graft | Biliary Pancreatitis |
| Liver disease | Alcoholic Cirrhosis with alcoholic hepatitis biopsy | HCV and ALD ApoA1 2.61 g/L | ALD haptoglobin 3.32g/L. | Chronic HCV  ALT 542 IU/L | NAFLD  Lipase 706 IU/L |
| Hepatocellular insufficiency | Yes | No | No | No | No |
| Ascites | Yes | No | No | No | No |
| FibroTest | 0.78 True Positive | 0.32 True Negative | 0.70 False positive | 0.78 Undeterminate | 0.60 False positive |

**Additional File 5, Table S10: Low Apoa1 <=0.56**

| **6** | **330708** | **221858** | **253081** | **40963** | **57037** | **249615** |
| --- | --- | --- | --- | --- | --- | --- |
| **Factor** |  |  |  |  |  |  |
| Age (year) | 42 | 33 | 35 | 75 | 65 | 67 |
| Gender | F | M | F | M | F | M |
| Continent | Far East | Sub Saharan Africa | W Europe | W Europe | W Europe | W Europe |
| Apolipoprotein A1 g/L | 0.12 | 0.39 | 0.32 | 0.16 | 0.13 | 0.40 |
| Proteins (g/L) | 54 | 88 | 50 | 44 | 44 | 64 |
| Main diagnosis | Hepatitis B and severe alcoholic hepatitis | Hepatitis B AgHBe+ HBVDNA>10^8^ HIV treated | Alcoholic cirrhosis undernutrition | Alcoholic liver disease | Liver metastasis undernutrition | Hepatocellular carcinoma Cirrhosis HCV |
| Liver disease | Cirrhosis | Biopsy F0A0 Immuno tolerance | Biopsy F4 Alcoholic hepatitis | ALD cirrhosis Death from ESLD | Biopsy: epidermoid carcinoma | Cirrhosis HCV G1 and alcohol Peritoneal metastasis Death ESLD |
| Hepatocellular insufficiency | yes Child C | no | yes Child C | Yes Child C | No | Yes Child C |
| Ascites | yes | no | yes | yes | No | Yes |
| Initial FT/METAVIR | 0.88 True Positive | 0.68 False Positive | 0.71 F3 True Positive | 0.85 F4 True Positive | 0.86 False Positive | 0.69 F3 True Positive |

| **8** | **352716** | **231508** | **182506** | **13369** | **359374** | **19222/19181 x2** | **50302** |
| --- | --- | --- | --- | --- | --- | --- | --- |
| Factor | #7 | #8 | #9 |  |  |  |  |
| Age (year) | 53 | 32 | 75 | 42 | 62 | 53 | 45 |
| Gender | M | F | F | M | M | M | M |
| Continent | W Europe | W Europe | W Europe | S Europe | S Europe | W Europe | W Europe |
| Apolipoprotein A1 g/L | 0.39 | 0.31 | 0.34 (1.57) | 0.40 (0.49/0.34 1 month before) | 0.12 | 0.35 ( 0.33/0.80) | 0.18 |
| Proteins (g/L) | 38 | 59 | 57 | 60 | 62 | - | 58 |
| Main diagnosis | ALD hemorrhage | Pulmonary abscess | Cirrhosis HCV | ALD  Tuberculosis | ALD | Hemorrhage varices ALD Child C | Severe Pancreatitis  ALD |
| Liver disease | Cirrhosis | HCV | Cirrhosis Biopsy F4 | Alcoholic cirrhosis | Alcoholic cirrhosis  Biopsy F4 | Alcoholic cirrhosis | ALD |
| Hepatocellular insufficiency | yes | No | Yes | Yes | Yes | Yes | Yes |
| Ascites | yes | No | Yes Large (14 g /L) Spontaneous Bacterial peritonitis | Yes (25g/L) Spontaneous Bacterial peritonitis | No | Yes | Yes |
| Initial FT/METAVIR | 0.62 True Positive | 0.53 True Positive | 0.55 True Positive | 0.64 True positive (0.77/0.81) | 0.83 True positive | 0.62 True positive (0.76/0.44) | 0.78 Indeterminate |

| **3** | **7640/7412** | **12790** |
| --- | --- | --- |
| **Factor** |  |  |
| Age (year) | 54 | 45 |
| Gender | M | M |
| Continent | W Europe | Asian |
| Apolipoprotein A1 g/L | 0.22 (0.15) | 0.29 |
| Proteins (g/L) | 65 | 67 |
| Main diagnosis | Variceal bleeding Cirrhosis HCV, ALD | Sepsis HCC, HBV |
| Liver disease | Cirrhosis HCV, ALD  Varices | Cirrhosis HBV, HCC  Death |
| Hepatocellular insufficiency | Yes | Yes |
| Ascites | Yes | No |
| Initial FT/METAVIR | 0.82 True positive (0.77) | 0.61 True positive |

**Additional file 5, Table S11: High A2M >= 5.90**

|  | **244558** | **55179** | **255226** | **117196 x2** | **250665** | **170327** | **36003** |
| --- | --- | --- | --- | --- | --- | --- | --- |
| **Factor** |  |  |  |  |  |  |  |
| Age (year) | 53 | 45 | 51 | 59 | 42 | 58 | 37 |
| Gender | M | M | M | M | M | M | M |
| Continent | Sub Saharian Africa | Sub Saharian Africa | W Europe | W Europe | W Europe | W Europe | W Europe |
| Alpha2 macroglobulin g/L | 6.02 (2.5/2.26/4.43/4.81/4.37) | 5.96 | 6.61 (2.78/3.78/5.12) | 6.7 (5.20/4.81/5.36/ 6.2) | 7.06 (6.26/5.87) | 6.02 | 6.35 (4.71/5.55) |
| Proteins (g/L) | 73 | 82 | NA | NA | NA | NA | 79 |
| Main diagnosis | Hepatitis C  Renal transplanted  **Kaposi chemotherapy**  LB 8mm, 4 fragments, F2A1, steatosis 10% | Hepatitis C, HIV  ALD | Hepatitis C, HIV | NASH | Hepatitis C, hepatitis B, HIV | Hepatitis B (cirrhosis)  Hepato-renal polykystosis (renal transplanted)  Type 2 diabetes | Hepatitis C, HIV |
| Liver disease | Hepatitis C | Hepatitis C, HIV, ALD  LB F4A0 (F1A2,/F4A1) | Hepatitis C, HIV  LSM 46.4kPa,  LB F1A2/F4A2/F4A2  Esophageal varices | NASH  LB F3, steatosis 60%/ F3 steatosis 50%,  LSM 34.8kPa | Hepatitis C, hepatitis B, HIV.  LB F2A2/F1A1 | Hepatitis B (cirrhosis Child A) and hepatic polykystosis  septicemia sigmoiditis | Hepatitis C, HIV  Cirrhosis Child A |
| Hepatocellular insufficiency | No | No | No | No | No | No | No |
| Ascites | No | No | No | No | No | No | No |
| FibroTest | 0.64 True positive (0.81/0.85/0.45) | 0.82 True positive. | 0.91 True positive (0.61/0.95)  . | 0.89 True positive (0.84/0.82/0.82/0.89) | 0.84 True positive (0.91/0.79) | 0.63 True positive | 0.78 True positive (0.89/0.81) |

|  | **79038** | **256364** | **248022** | **200324** | **68530** | **217817 x3** |
| --- | --- | --- | --- | --- | --- | --- |
| **Factor** |  |  |  |  |  |  |
| Age (year) | 21 | 46 | 52 | 58 | 45 | 48 |
| Gender | M | M | M | M | M | M |
| Continent | Sub Saharian Africa | N African | N African | W Europe | W Europe | W Europe |
| Alpha2 macroglobulin g/L | 5.99 (3.63/3.31/3.42) | 6.71 | 6.46 (5.36/4.82) | 7.45 | 6.26 (3.44 4 years after) | 7.13 (1.74/3.8/4.3/5.66/6.52/5.99/4.98/6.69) |
| Proteins (g/L) | 36 | NA | 87 | 84 | 84 | 79 |
| Main diagnosis | HBV Renal failure | HCV HIV | HCV HIV | HCV HIV | HCV  Vascularitis | HCV HIV |
| Liver disease | HBV | HCV HIV  Biopsy F3 | HCV HIV  Biopsy F2  LSM 11.7/12.3/4.5kPa | HCV HIV  LSM 4.4kPa | HCV | HCV HIV  LSM 5.9/8.6kPa |
| Hepatocellular insufficiency | No | No | No | No | No | No |
| Ascites | No | No | No | No | No | No |
| FibroTest | 0.48 True positive (0.92/0.67/0.70) | 0.90 True positive | 0.83 True positive (0.78/0.86) | 0.84 Indeterminate | 0.45  Indeterminate (0.26) | 0.90 True Positive (0.47/0.70/0.7/0.87/0.89/0.79/0.66/0.85) |

**Additional file 5, Table S12: Clinical characteristics of patients with high risk profile identified in reference center: Low A2M (<=0.80g/L)**

| **6 included** | **325155*** | **330711** | **339820** | **345241** | **350577** | **352150** | **352168** |
| --- | --- | --- | --- | --- | --- | --- | --- |
| **Factor** |  |  |  |  |  |  |  |
| Age (year) | 70 | 60 | 60 | 50 | 50 | 55 | 63 |
| Gender | M | M | M | M | M | M | M |
| Continent | W Europe | W Europe | W Europe | E Europe | W Europe | W Europe | Far East |
| Alpha2 macro g/L | 0.72 | 0.67 | 0.36 | 0.69 | 0.79 | 0.70 | 0.49 |
| Proteins (g/L) | 122 | 57 | 22 | 49 | 83 | 60 | 52 |
| Main diagnosis | Macrophage activation syndrome ferritinin 14800 IU/l  myeloma | Hemorrhage HNR biopsy portal hypertension LSM 4kPa Colon cancer | Hemorrhage gastric ulcer Undernutrition | Undernutrition | Cardiac insufficiency liver sinusoidal dilatation biopsy F1-F2 | Duodenal Ulcer | Gastric Ulcer hemorrhage |
| Liver disease | Acute Hepatitis | No | No | No | No | No | No |
| Hepatocellular insufficiency | yes | no | no | no | no | no | no |
| Ascites | no | no | no | no | High proteins 58g/L | no | no |
| Final FibroTest Interpretation | 0.49 False positive | 0.04True negative | 0.05 True negative | 0.14 True negative | 0.37 True negative | 0.05 True negative | 0.09 True negative |

* excluded also for ALT 2059 IU/L

| **7** | **47119** | **12500** | **39724** | **288699** | **119238** | **85590** | **283625** |
| --- | --- | --- | --- | --- | --- | --- | --- |
| **Factor** |  |  |  |  |  |  |  |
| Age (year) | 51 | 63 | 47 | 42 | 61 | 53 | 62 |
| Gender | M | F | M | M | M | M | M |
| Continent | W Europe | W Europe | W Europe | W Europe | W Europe | W Europe | North Africa |
| Alpha2 macro g/L | 0.71 | 0.69 | 0.61 | 0.76 | 0.77 | 0.71 (1.41/0.68/ 0.7) | 0.60 (0.43/1.40/1.51) |
| Proteins (g/L) | NA | 53 | 34 | 34 | 47 | 70 | 50 (81) |
| Main diagnosis | HIV HCV | ALD/NAFLD renal insufficiency/diabetes | ALD/hemorrhage | ALD/hemorrhage/HCV/HIV undernutrition | Hemorrhage/ALD/ undernutrition | NAFLD | Hemorrhage duodenal ulcer |
| Liver disease | 5.1kPa | cirrhosis | cirrhosis | cirrhosis | Cirrhosis | NAFLD | ALD |
| Hepatocellular insufficiency | No | Yes | Yes Death | Yes | Yes | No | No |
| Ascites | No | Yes 15g proteins/L | Yes | No | Yes 8g proteins/L | No | No |
| FibroTest Final Interpretation | 0.08 True negative | 0.62 True positive | 0.54 True positive | 0.63 True positive | 0.37 False negative | 0.10 True negative | 0.04 True negative |

| **7** | **274110** | **6679** | **274829** | **34313** | **7381** | **21182** | **24032** |
| --- | --- | --- | --- | --- | --- | --- | --- |
| **Factor** |  |  |  |  |  |  |  |
| Age (year) | 55 | 60 | 50 | 60 | 39 | 51 | 21 |
| Gender | M | M | M | M | M | M | M |
| Continent | W Europe | N Africa | W Europe | W Europe | W Europe | Far East | Sub Saharan Africa |
| Alpha2 macro g/L | 0.77 (1.74) | 0.53 (0.38/0.78) | 0.78 (0.90/0.85/0.95) | 0.68 | 0.61 | 0.77 | 0.59 (1.91) |
| Proteins (g/L) | 44 | 48 | 74 | 50 | 71 | 61 | 54 (92) |
| Main diagnosis | Hemorrhage oesophagitis  Undernutrition | Hemorrhage duodenal ulcer | NAFLD obesity | Hemorrhage duodenal ulcer | ALD | Hemorrhage duodenal ulcer | Sepsis hemorrhage oesophageal varices |
| Liver disease | ALD F1 biopsy | ALD | NAFLD biopsies F0/F1 LSM 4.8/4.0 kPa | No | ALD biopsy F3 | ALD pancreatitis | Autoimmune cirrhosis biopsy F4 |
| Hepatocellular insufficiency | No | No | No | No | No | No | Yes |
| Ascites | No | No | No | No | No | No | Yes |
| FibroTest Final Interpretation | 0.07 True negative | 0.04 True negative | 0.07 True negative | 0.17 True negative | 0.07 False negative | 0.17 True Negative | 0.20 False negative |

| **7** | **225648** | **41567** | **49346** | **86755** | **78578** | **290942** | **52308** |
| --- | --- | --- | --- | --- | --- | --- | --- |
| **Factor** |  |  |  |  |  |  |  |
| Age (year) | 20 | 49 | 46 | 27 | 42 | 48 | 49 |
| Gender | M | M | M | M | M | M | M |
| Continent | Sub Saharan Africa | W Europe | W Europe | W Europe |  | W Europe |  |
| Alpha2 macro g/L | 0.63 | 0.77 | 0.65 | 0.77 (1.04/1.25) | 0.79 | 0.36 | 0.79 |
| Proteins (g/L) | 93 | 37 | 61 | 86 |  |  |  |
| Main diagnosis | Sepsis Drepanocytosis bone marrow graft cardiac insufficiencyDeath | Hemorrhage gastric varices ALD cirrhosis | Hemorrhage oesophagitis | LSM 7.9/7.1 kPa |  |  |  |
| Liver disease | HCV LSM 3.9kPa | ALD cirrhosis | ALD | HCV |  |  |  |
| Hepatocellular insufficiency | No | Yes | No | No |  |  |  |
| Ascites | No | Yes | No | No |  |  |  |
| FibroTest Final Interpretation | 0.17 True Negative | 0.34 False negative | 0.05 True negative | 0.14 False negative | 0.11 TN | 0.03 TN | 0.12 TN |

| **5** | **298391/**161292 | **9656** | **20536** | **298399** |
| --- | --- | --- | --- | --- |
| **Factor** |  |  |  |  |
| Age (year) | 45 | 39 | 81 | 44 |
| Gender | M | M | M | M |
| Continent | North Africa | W Europe | W Europe | W Europe |
| Alpha2 macro g/L | 0.65 (0.70) | 0.76 | 0.76 | 0.78 |
| Proteins (g/L) |  |  |  |  |
| Main diagnosis |  |  |  |  |
| Liver disease |  |  |  |  |
| Hepatocellular insufficiency |  |  |  |  |
| Ascites |  |  |  |  |
| FibroTest Final Interpretation | 0.08 TN | 0.10 TN | 0.14 TN | 0.25 TN |

|  | **324114** | **247553** | **180963** | **150014** | **42039** | **258005 x2** | **217817** |
| --- | --- | --- | --- | --- | --- | --- | --- |
| **Factor** |  |  |  |  |  |  |  |
| Age (year) | 41 | 44 | 59 | 43 | 47 | 76 | 48 |
| Gender | M | M | F | F | F | F | M |
| Continent | North Africa | North Africa | Sub Saharian Africa | W Europe | North Africa | Asia | W Europe |
| Alpha2 macroglobulin g/L | 6.12 | 6.12 (5.09) | 6.13 | 6.35 (5.09) | 6.64 | 6.23 (5.94/5.90) | 7.13 |
| Proteins (g/L) | 81 | 81 (42) | NA | NA | NA | 60 | NA |
| Main diagnosis | HCV, HIV, ALD | HCV, HIV | HCV, HIV, ALD | HCV, HIV | HCV, HIV | HCV NAFLD | HC, HIV |
| Liver disease | F3 biopsy LSM 27.0kPa | HCV  LSM 26.6kPa | Hepatitis C, HIV, ALD  Biopsy F3 | Hepatitis C, HIV  LSM 10.8kPa  Biopsy F3 | Hepatitis C, HIV  Biopsy F2 | Hepatitis C Type 2 Diabetes  AIH LSM 12.5kPa | Hepatitis C, HIV  Biopsy F2 hypertension at ultrasound. |
| Hepatocellular insufficiency | no | No | No | No | No | No | No |

| Ascites | no | No | No | No | No | No | No |
| --- | --- | --- | --- | --- | --- | --- | --- |
| FibroTest Conclusion | 0.84 True positive | 0.85 True positive (0.86) | 0.83 True positive | 0.91 True positive (0.83) | 0.56 True positive | 0.88 True positive (0.87/0.80) | 0.90 True positive |

**Additional File 5, Table S13: High Apoa1 >= 2.50**

| **5** | **76103** | **174693** | **59944** | **244951** | **296984** |
| --- | --- | --- | --- | --- | --- |
| **Factor** |  |  |  |  |  |
| Age (year) | 52 | 53 | 45 | 37 | 77 |
| Gender | M | M | M | M | F |
| Continent | W Europe | Sub-Saharan Africa | Sub-Saharan Africa | W Europe | W Europe |
| Apolipoprotein A1 g/L | 3.11 | 2.88 (2.02) | 2.66 | 3.29 | 2.83 |
| Proteins (g/L) | 70 | NA | 72 | NA | 77 |
| Main diagnosis | Hepatitis C | Hepatitis C | Hepatitis C | ALD | HCV |
| Liver disease | HCV Biopsy F1 | HCV HIV | HCV Biopsy F0 | ALD HBV Delta | HCV Biopsy F2 LSM 4.5 /5.1/6.8/7.0 kPa |
| Hepatocellular insufficiency | No | No | No | No | No |
| Ascites | No | No | No | No | No |
| FT/METAVIR | X True negative | X Indeterminate | x True negative | 0.33 Indeterminate | x False negative |

| **9** | **180960** | **26800 x2 27550** | **252575** | **288716** | **98324** | **117652 x2 330939** | **247846** |
| --- | --- | --- | --- | --- | --- | --- | --- |
| **Factor** |  |  |  |  |  |  |  |
| **Age (year)** | 55 | 46 | 70 | 63 | 50 | 56 | 75 |
| **Gender** | F | F | F | M | F | F | F |
| **Continent** | W Europe | W Europe | W Europe | Asia | Sub-Saharan Africa | W Europe | W Europe |
| **Apolipoprotein A1 g/L** | 2.69 | 2.94 (2.16/2.61) | 2.77 (2.5) | 2.60 (2.79/2.61) | 2.60 | 2.82 (2.64/2.23/2.96) | 4.12 (2.01/1.88) |
| **Proteins (g/L)** | 64 | 73 | 78 | 63 | 77 | 78 | 61 |
| **Main diagnosis** | Hepatitis C | ALD | Hepatitis C | Hepatitis B | Hepatitis C | Hepatitis C | Hepatitis C |
| **Liver disease** | HCV Biopsy A1F1 Steatosis 95% A1F1 | ALD renal pancreas transplanted  **Biopsy** F0 | HCV AIH  LSM 5.9kPa | Hepatitis B | Hepatitis C  Biopsy F1  LSM 4.0kPa (not applicable) | Hepatitis C G1  Biopsy F0  LSM 7.4kPa | Hepatitis C Biopsy F1  LSM 23.8/14.1kPa |
| **Hepatocellular insufficiency** | No | No | No | No | No | No | No |
| **Ascites** | No | No | No | No | No | No | No |
| **FT/METAVIR** | 0.29 True negative | 0.29 True negative. | 0.32 True negative (0.29) | 0.22 True negative (0.16/0.24) | 0.34 True negative. | 0.14 True negative (0.12/0.22/0.13) | 0.08 True negative (0.48/0.45). |

| **9** | **255929/244561 x2** | **174693/357136 x2** | **266875** | **244559** | **300032** | **261597** | **109157** |
| --- | --- | --- | --- | --- | --- | --- | --- |
| **Factor** |  |  |  |  |  |  |  |
| **Age (year)** | 37 | 55 | 30 | 54 | 78 | 39 |  |
| **Gender** | M | M | F | M | F | F | F |
| **Continent** | Sub Saharan Africa | Sub Saharan Africa | Sub Saharan Africa | Sub Saharan Africa | W Europe | Sub Saharan Africa | W Europe |
| **Apolipoprotein A1 g/L** | 3.14 (2.31/3.29) | 2.88 (2.43/2.02/2.69) | 2.75 (2.02) | 2.75 (1.85) | 2.65 (2.02/1.34/2.68/2.15/2.32/2.34) | 3.03 (3.04) | 2.99 |
| **Proteins (g/L)** | - | - | - | 78 | 75 | - | 74 |
| **Main diagnosis** | Hepatitis B, ALD | Hepatitis B, HIV | Hepatitis C, HIV | Hepatitis B | Hepatitis C | Hepatitis C, HIV, Chronic renal failure, diabetes. | ALD, Depression |
| **Liver disease** | Hepatitis B, ALD Biopsy F1. LSM 3.9kPa | Hepatitis B, HIV. | Hepatitis C, HIV, LSM 7.3kPa Biopsy F1 (7yr before) | Hepatitis B cardiac transplantation Biopsy F1 | Hepatitis C  LSM 9.9/11.6/5.2kPa | Hepatitis C, HIV. LSM 6.8kPa | ALD |
| **Hepatocellular insufficiency** | No | No | No | No | No | No | No |
| **Ascites** | No | No | No | No | No | No | No |
| **FT/METAVIR** | 0.25 True negative (0.15/0.33). | 0.26 True negative (0.33/0.41/0.33). | 0.53 True positive (0.59) | 0.17 True negative (0.26) | 0.42 False negative (0.70/0.78/0.47/0.75 /0.56/0.35) | 0.12 True negative (0.07) | 0.18 True negative |

| **6** | **44861** | **59944** | **91994** | **210987** | **252428** | **256369** |
| --- | --- | --- | --- | --- | --- | --- |
| **Factor** |  |  |  |  |  |  |
| **Age (year)** | 59 | 45 | 43 | 58 | 36 | 57 |
| **Gender** | M | M | F | M | M | F |
| **Continent** | W Europe | Sub Saharan Africa | Sub Saharan Africa | Sub Saharan Africa | W Europe | W Europe |
| **Apolipoprotein A1 g/L** | 2.58 | 2.66 | 3.01 (1.97/2.11/2.12) | 2.70 (1.72/1.90) | 2.88 | 2.69 (1.74/1.87) |
| **Proteins (g/L)** | 88 | 72 | 73 | 79 | 79 | NA |
| **Main diagnosis** | ALD | Hepatitis C | Hepatitis C and B  Diabetes | Hepatitis C | ALD | Hepatitis C |
| **Liver disease** | ALD | Hepatitis C  LB F0A1 | Hepatitis C B  LSM 5.6kPa | Hepatitis C  LSM 8.6 kPa | ALD  LSM 4.7kPa | Hepatitis C  LSM 7.9/6.2kPa |
| **Hepatocellular insufficiency** | No | No | No | No | No | No |
| **Ascites** | No | No | No | No | No | No |
| **FT/METAVIR** | 0.29 True negative | 0.22 True negative | 0.24 True negative (0.18/0.18/0.30) | 0.44 False negative (0.68/0.59) | 0.16 True negative | 0.18 False negative (0.45/0.54/ 0.82) |

| **9** | **102379/279531/316153/356837** | **116712** | **151591** | **23309** | **84590** | **88090** |
| --- | --- | --- | --- | --- | --- | --- |
| **Factor** |  |  |  |  |  |  |
| **Age (year)** | 54 | 58 | 64 | 43 | 45 | 75 |
| **Gender** | F | F | F | M | F | F |
| **Continent** | W Europe | W Europe | W Europe | **W Europe** | Sub Saharan Africa | W Europe |
| **Apolipoprotein A1 g/L** | 2.88g/L (2.69g/L 1year before, 2.64/2.79/2.82g/L 3/3/4 years after) | 2.58g/L (2.37/2.13/2.12g/L 3/2/1 years before | 2.6g/L | 2.69g/L (2.45/2.42/0.32 1/2/3 year after) | 3.10 (2.01) | 2.68 (2.02/1.34/2.15/2.39/2.65/2.34) |
| **Proteins (g/L)** | 76 | - | 61 | 77 | 82 |  |
| **Main diagnosis** | Hepatitis B  Renal transplanted  Biopsy F3  LSM 8.3/6.7 kPa | Hepatitis B AIH  Biopsy F1 | DILI Heart-lung transplanted  Renal Failure | Hepatitis B | ALD | HCV |
| **Liver disease** | Hepatitis B | Hepatitis B AIH | LSM 6.6kPa  Biopsy F1 | Hepatitis B | ALD  5.4kPa OV grade1 | HCV  LSM 9.9 (11.6/5.2kPa) |
| **Hepatocellular insufficiency** | No | No | No | No | No | No |
| **Ascites** | No | No | No | No | No | No |
| **FT/METAVIR** | 0.26 True negative (0.22/0.19/0.12/0.10) | 0.26 True negative (0.29/0.49/0.43) | 0.30 True negative | 0.26 True negative (0.25/0.17/0.32) | 0.35 Indeterminate (0.11) | 0.47 Indeterminate (0.70/0.78/0.75 /0.56/0.42/0.35). |
